# New understanding of the molecular pathogenesis and therapeutic progress of hepatocellular carcinoma

**DOI:** 10.3389/or.2026.1763632

**Published:** 2026-04-17

**Authors:** Wei-Jun Wang, Hong-Xia Yuan, Jin-Zhen Xu, Xing-Dong Wang, Guang-Wei Na, Ke-Ji He, Wen-Ping Sun, Rui Li, Jing Che, Yong-Sheng Cheng

**Affiliations:** Gansu Provincial Cancer Hospital, Lanzhou, Gansu, China

**Keywords:** hepatocellular carcinoma, staging, molecular pathogenesis, signal pathway, advanced therapy

## Abstract

Hepatocellular carcinoma (HCC) is a lethal malignancy with strong ties to chronic liver diseases and metabolic risks. Its pathogenesis involves complex biological processes where disrupted tumor suppressor genes and activated oncogenes drive aberrant signaling pathways and cellular differentiation. Since most patients are diagnosed at advanced stages, only a minority qualify for curative therapies. Although significant therapeutic advances have been made, the overall prognosis remains poor, largely due to high rates of recurrence and metastasis. Therefore, a deeper understanding of HCC’s molecular mechanisms is crucial for developing effective targeted treatments. This review summarizes current insights into HCC pathogenesis and systematically elaborates on contemporary therapies, discussing their respective advantages and limitations.

## 1 Background

Hepatocellular carcinoma (HCC), the predominant form of primary liver cancer worldwide, arises from the malignant transformation of hepatocytes and is characterized by its high aggressiveness (1–3). According to GLOBOCAN 2022, HCC is responsible for approximately 865,000 incident cases and 758,000 deaths worldwide, positioning it as the sixth most frequently diagnosed malignancy and the third most common cause of cancer-related deaths (4,5). Notably, epidemiological data reveal a concerning upwards trajectory in both HCC incidence and mortality rates. The incidence of HCC is particularly high in northern and western Africa and in eastern and southeastern Asia. China accounts for nearly 50% of global HCC cases (6). The dismal outcomes reflect both aggressive tumor biology and the fact that most patients present at advanced stages, with curative options applicable to only 15% of cases (7–11).

HCC has emerged as a critical global health challenge, primarily in individuals with chronic liver disease (CLD), such as fatty liver disease or cirrhosis. Major risk factors include alcohol abuse, hepatitis B or C infection, and metabolic disorders such as diabetes mellitus (12–14). Less common risk factors include hereditary hemochromatosis (15), acute intermittent porphyria (16), alpha-1 antitrypsin deficiency (17), autoimmune hepatitis, and environmental carcinogens such as aflatoxin (18).

The early stage of HCC can usually be treated by potential curative methods, such as surgical resection, including hepatectomy, radiofrequency ablation and liver transplantation (19). However, the discovery and treatment of HCC still face significant challenges. With over 60% of patients presenting at intermediate or advanced stages, surgical eligibility is limited to 10%–20% of cases, further constrained by underlying cirrhosis. Additionally, there is a notable gap in postoperative adjuvant therapy. Immune checkpoint inhibitors, such as PD-1/PD-L1 antibodies, are effective in only 30% of patients, and both primary and secondary drug resistance are common (20).

In this article, we provide a concise summary of the current development status and classification of HCC. We thoroughly explore the pathogenic mechanisms underlying HCC and detail the current treatment methods, along with their advantages and disadvantages. Through this review, readers will gain a deeper understanding of the development process of HCC and anticipate new directions for future treatment and research in this area.

## 2 Diagnosis and staging of HCC

### 2.1 Barcelona clinical liver carcinoma (BCLC) system

The Barcelona Clinical Liver Carcinoma (BCLC) system is one of the most widely used staging systems (21).The BCLC staging system provides both tumor staging and treatment recommendations (Table 1). However, for advanced diseases characterized by multiple heterogeneous disease phenotypes and varying prognoses, it solely recommends sorafenib as the treatment option (22).

**TABLE 1:** The BCLC system of HCC.

Stage	Tumor characteristics	Liver function (child-pugh)	PS Score	Treatment recommendations	Prognosis
0	Single tumor ≤2 cm	Class A	0 Fully active	Surgical resection/ablation/liver transplant	5-year survival >70%
A	Single tumor or ≤3 nodules, each ≤3 cm	Class A-B	0 Fully active	Liver transplant/resection/ablation	5-year survival 60%–70%
B	Multinodular tumors without vascular invasion	Class A-B	0 Fully active	Transarterial chemoembolization (TACE)/systemic therapy	Median survival ∼2 years
C	Vascular invasion or extrahepatic spread	Class A-B	1–2 restricted activity	Targeted/immunotherapy (e.g., lenvatinib)	Median survival 6–12 months
D	Severe liver dysfunction (child-pugh C)	Class C	≥3 Disabled or bedridden	Palliative care	Survival <3 months

### 2.2 Tumor node metastasis (TNM) staging system

The TNM staging system is a standard classification system for describing the anatomical range of malignant tumors. The T parameter assesses the local characteristics of the primary tumor, including tumor number, tumor size, and vascular invasion (Table 2), the N parameter reflects the regional lymph nodes (Table 3), while the M parameter is used to determine the presence of distant metastasis (Table 4) (20,23,24).

**TABLE 2:** The TNM staging system of the T parameter.

T (primary tumor)
T1: Single tumor without vascular invasion
T2: Single tumor with vascular invasion, or multiple tumors ≤5 cm
T3: Multiple tumors >5 cm, or invasion of major branches of the portal/hepatic veins
T4: Invasion of adjacent organs or penetration of the visceral peritoneum

**TABLE 3:** The TNM staging system of the N parameter.

N (lymph node metastasis)
N0: No regional lymph node metastasis
N1: Regional lymph node metastasis

**TABLE 4:** The TNM staging system of the M parameter.

M (distant metastasis)
M0: No distant metastasis M1: Distant metastasis Stage grouping Stage IA: T1N0M0 Stage IB: T2N0M0 Stage II: T3N0M0 Stage IIIA: T4N0M0 Stage IIIB: Any T with N1M0

### 2.3 Okuda staging system

The Okuda staging system was the first to consider both tumor and liver function parameters. There are three stages on the basis of tumor size, presence of ascites, jaundice and albumin and bilirubin levels. However, this system is not widely used because it does not consider multifocal or vascular infiltration of the tumor (20,25).

## 3 The molecular pathogenesis of HCC

The molecular pathogenesis of HCC is driven by a multilayered regulatory network integrating genetic alterations, epigenetic dysregulation, oncogenic signaling pathways, and tumor microenvironmental interactions. In this conceptual framework, genetic mutations represent initiating events, while epigenetic modifications reshape transcriptional programs that reinforce oncogenic signaling cascades. These signaling pathways subsequently influence tumor cell proliferation, metabolism, and survival, while simultaneously interacting with the tumor microenvironment (TME) to establish an immunosuppressive niche that promotes tumor progression. This hierarchical yet dynamic model provides an integrative perspective for understanding the complex biology of HCC.

### 3.1 Genetic mutations in HCC

#### 3.1.1 TERE gene mutations

Telomerase, a key reverse transcriptase that maintains telomere stability, slows the process of cellular senescence by inhibiting telomere shortening. In adult liver tissues, most hepatocytes undergo persistent telomere shortening due to telomerase silencing. This promotes hepatocyte senescence and contributes to hepatic fibrosis and cirrhosis (26–28). Transitional epithelial response gene 1 (TERT) is encoded by the TERT gene, and the mechanisms of its reactivation in HCC include somatic telomerase promoter mutations (29), amplification at the telomerase DNA level (30), viral insertion into the telomerase gene (31–34), and alternative telomere lengthening (35,36).

#### 3.1.2 TP53 gene mutations

The R249S mutation in the TP53 gene is more common in HCC and is associated with aflatoxin B1 and HBV infection. This mutation may be the result of aflatoxin B1 causing a specific G to T translocation mutation in the TP53 gene. Mutations in the TP53 gene are associated with high chromosomal instability and rarely occur in conjunction with mutations in the catenin beta 1 (CTNNB1) gene. In addition, mutations in the TP53 gene have been associated with alterations in other cell cycle-related genes, such as the cyclin-dependent kinase inhibitor 2A(CDKN2A) gene, HBV insertions in the CCNE1 gene, amplification of the CCND1/FGF19 locus, and somatic mutations in the retinoblastoma 1 (RB1) gene (37–39).

From a clinical perspective, TP53-mutated HCC is frequently associated with aggressive tumor behavior, higher recurrence rates, and unfavorable prognosis, suggesting that TP53 status may serve as a potential prognostic biomarker in HCC (40–42).

#### 3.1.3 Wnt/β-catenin pathway-related mutations

Activating mutations in the CTNNB1 gene encoding β-catenin are among the most common genetic alterations in HCC and frequently co-occur with TERT promoter mutations, suggesting a synergistic interaction between Wnt/β-catenin signaling and telomerase activation during hepatocarcinogenesis (28,32,43). Approximately 18.5% of HCC cases harbor CTNNB1 mutations, of which nearly 75% are missense substitutions, and the mutation frequency is closely associated with etiological factors such as viral infection and metabolic liver disease (44,45). In addition to CTNNB1, mutations in other components of the Wnt pathway also contribute to pathway activation; for example, AXIN1 is mutated in approximately 8% of HCCs, including 30% missense and 37.5% nonsense substitutions (45).

Experimental models further support the oncogenic role of Wnt/β-catenin signaling in liver tumorigenesis. Co-expression of activated β-catenin with oncogenic drivers such as mesenchymal-epithelial transition (MET) or kirsten rat sarcoma viral oncogene homolog (KRAS) can induce liver tumors that closely resemble human HCC. Likewise, liver-specific adenomatous polyposis coli (APC) knockout mice spontaneously develop HCC characterized by constitutive β-catenin activation (46).

Clinically, tumors with β-catenin activation frequently exhibit an immune-excluded tumor microenvironment and demonstrate reduced responsiveness to immune checkpoint inhibitors, indicating that alterations in the Wnt/β-catenin pathway may serve as important biomarkers for patient stratification and therapeutic decision-making in HCC (47,48).

#### 3.1.4 Oxidative stress pathway-related mutations

Although mutations in oxidative stress pathway genes are relatively uncommon in HCC, their dysregulation profoundly influences tumor behavior. Normally, the nuclear factor erythroid 2-like 2 (NFE2L2) is bound and targeted for degradation by its repressor kelch-like ECH-associated protein 1 (KEAP1), whereas oxidative stress induces its release, nuclear translocation, and activation of cytoprotective genes. In HCC, inactivating KEAP1 mutations or activating NFE2L2 alterations disrupt this homeostasis, resulting in constitutive NFE2L2-driven transcription. This sustained activation not only augments antioxidant capacity and cell survival (49).

#### 3.1.5 Chromatin remodeling-associated mutations

Both AT-rich interactive domain-containing protein 1A (ARID1A) and AT-rich interactive domain-containing protein 2 (ARID2), are recurrently mutated in HCC and function as tumor suppressors, in part through their roles in DNA repair and genomic integrity. The hepatitis B virus X protein (HBx) contributes to hepatocarcinogenesis by suppressing ARID2 expression, thereby disrupting growth regulatory homeostasis and promoting tumor development (39,50–52). HBV genotypes B, C and A1, core promoter/preS mutations, HBX truncations, and spliced pgRNAs collectively drive HCC progression (53). Bromodomain-containing protein 7 (BRD7), is frequently truncated in HCC. Its loss-of-function mutations are enriched in HBV-HCC and correlate with tumor size/stage and survival. HCV infection downregulates BRD7, promoting HCC proliferation (34,52,54–57).

#### 3.1.6 RB1 pathway-related mutations

RB1 is a key tumor suppressor that regulates the G1/S transition of the cell cycle by controlling E2F transcription factors (58–60). Loss-of-function mutations or deletions of RB1 disrupt cell-cycle checkpoints and promote uncontrolled proliferation in HCC. Ribosomal protein L22 (RPL22), has also been reported to harbor multiple mutation types including SNVs, indels, structural variants, and copy number variations in HCC (61,62).

#### 3.1.7 Other gene mutations

**FIGURE 1:**
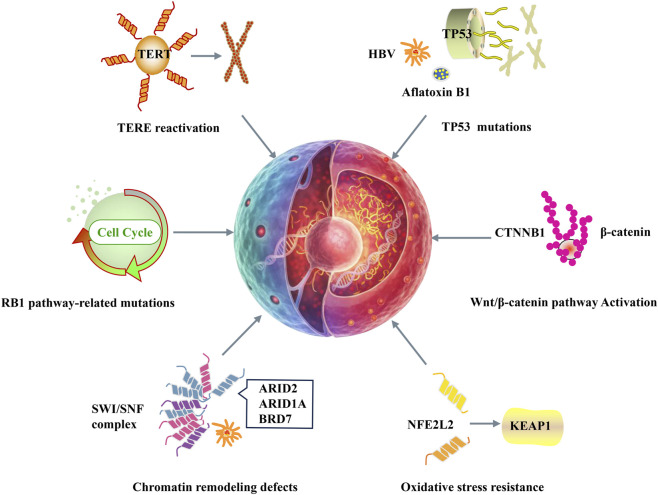
Genetic mutations in HCC. TERT activation, HBV, aflatoxin B1, and TP53 mutations drive HCC. CTNNB1 activates Wnt/β-catenin to promote proliferation. SWI/SNF subunit mutations (ARID2, ARID1A, BRD7) disrupt chromatin remodeling. NFE2L2 activation and KEAP1 mutations enhance oxidative stress resistance. RB1 pathway mutations dysregulate the cell cycle.

Other commonly mutated genes include CDKN2A, SWI/SNF-related matrix-associated actin-dependent regulator of chromatin subfamily A member 2 (SMARCA2), hepatocyte growth factor (HGF), methionine adenosyltransferase 1A (MAT1A), and glycine n-methyltransferase (GNMT). Mutations in these genes may disrupt critical biological processes such as telomerase activity regulation, cell cycle control, chromatin remodeling, and cell proliferation (63,64). There are also numerous low-frequency mutated genes in HCC, including ARID1A, tuberous sclerosis complex 1/2 (TSC1/TSC2), ribosomal protein S6 kinase A3 (RPS6KA3), and myeloid/lymphoid or mixed-lineage leukemia 2 (MLL2). These genes play a significant role in the pathogenesis of HCC (65) (Figure 1).

### 3.2 Epigenetic modification

**FIGURE 2:**
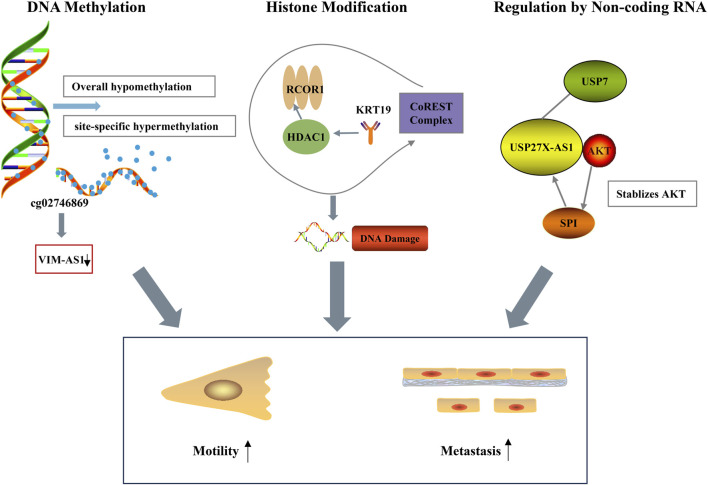
Epigenetic modifications in HCC. Global hypomethylation and site-specific hypermethylation downregulate VIM-AS1. Histone modifiers (RCOR1, KRT19, HDAC1, CoREST complex) induce DNA damage. USP27X-AS1 stabilizes AKT and regulates SPI to mediate signaling. These events enhance tumor cell motility and drive metastasis.

Epigenetic modification refers to the processes that regulate gene function and expression through chemical modifications without altering DNA sequences. Dysregulation of epigenetic mechanisms is critically involved in the initiation and progression of human malignancies (66,67). Research has identified numerous epigenetic regulators closely associated with HCC pathogenesis (2) (Figure 2).

#### 3.2.1 DNA methylation

DNA methylation is a key epigenetic regulatory mechanism and exhibits characteristic genome-wide hypomethylation accompanied by locus-specific hypermethylation in HCC (68). One representative example is CD147, a transmembrane glycoprotein frequently overexpressed in HCC. Hypomethylation of the CD147 promoter enhances the binding affinity of the transcription factor specificity protein 1 (SP1), thereby increasing CD147 transcriptional activity (69). Emerging evidence further indicates that the transforming growth factor-β (TGF-β) signaling pathway can transcriptionally activate CD147 by promoting the recruitment of SP1 and the DNA demethylation enzyme thymine DNA glycosylase (TDG) to the CD147 promoter. TDG participates in active DNA demethylation, leading to reduced promoter methylation levels and enhanced SP1-mediated transcriptional activation (70).

Functionally, the upregulation of CD147 contributes to tumor progression by stimulating the production of matrix metalloproteinases (MMP-2 and MMP-9), promoting extracellular matrix degradation and facilitating tumor cell invasion and metastasis. In addition, CD147 can enhance tumor–stromal interactions and angiogenesis, thereby accelerating HCC progression (71).

Beyond CD147, aberrant DNA methylation also regulates the expression of several oncogenic or tumor-promoting genes in HCC, including GTP binding protein 4 (GTPBP4), CREB regulated transcription coactivator 2 (CRTC2), colony stimulating factor 1 (CSF1), and CCAAT/enhancer binding protein beta (C/EBPβ) (72–75). Furthermore, recent studies have shown that hypermethylation of vimentin antisense RNA 1 (VIM-AS1) is closely correlated with unfavorable clinical outcomes in patients with HCC, suggesting that epigenetic alterations may serve as potential prognostic biomarkers (76).

#### 3.2.2 Histone modification

Histone modification constitutes a critical component of epigenetic regulation, primarily including acetylation, methylation, ubiquitination, and phosphorylation, which collectively modulate chromatin structure and transcriptional activity in HCC (2). Dysregulation of these modifications plays a pivotal role in hepatocarcinogenesis and tumor progression. For instance, keratin 19 (KRT19) promotes tumorigenesis by enhancing the interaction between histone deacetylase 1 (HDAC1) and REST corepressor 1 (RCOR1), thereby facilitating the assembly of the CoREST (Co-repressor of REST) complex and promoting transcriptional repression of tumor-suppressive genes (77,78).

Among histone methylation regulators, enhancer of zeste homolog 2 (EZH2) plays a particularly important role. EZH2 is the catalytic subunit of the polycomb repressive complex 2 (PRC2) and mediates trimethylation of histone H3 lysine 27 (H3K27me3). Overexpression of EZH2 in HCC results in aberrant accumulation of H3K27me3 and subsequent epigenetic silencing of several tumor suppressor genes, including CDKN2A, e-cadherin (CDH1), and PTEN, thereby promoting tumor cell proliferation, epithelial–mesenchymal transition (EMT), and metastatic potential (79–81).

In addition to methylation and acetylation, other histone modifications such as ubiquitination and phosphorylation are also frequently dysregulated in HCC. These alterations impair DNA damage response, disrupt cell-cycle regulation, and modulate transcriptional programs, collectively contributing to hepatocellular carcinogenesis and tumor progression (82). Importantly, epigenetic dysregulation also serves as a bridge between genetic mutations and downstream signaling pathways, as aberrant methylation, histone modifications, and ncRNA-mediated regulation can amplify oncogenic signaling networks such as PI3K/AKT/mTOR, JAK/STAT, and Wnt/β-catenin pathways, thereby reinforcing tumor growth and survival.

#### 3.2.3 Regulation by non-coding RNA (ncRNA)

NcRNAs play critical regulatory roles in HCC and participate in tumor initiation and progression through diverse molecular mechanisms. Among them, the long non-coding RNA USP27X antisense RNA 1 (USP27X-AS1) is significantly upregulated in HCC and has been associated with poor clinical prognosis. Mechanistically, USP27X-AS1 promotes the proliferation and metastasis of HCC by promoting the interaction between USP7 and AKT, reducing AKT polyubiquitination levels and enhancing AKT protein stability (83). Importantly, the interaction between USP27X-AS1 and AKT is mediated by specific RNA domains, which reflects the structural characteristics of long non-coding RNAs.

Epigenetic regulation also influences the function of certain lncRNAs in HCC. For example, the expression of VIM-AS1 is regulated by CpG methylation, and its hypermethylation is associated with poor prognosis in HCC. Downregulation of VIM-AS1 promotes tumor progression by facilitating the binding of insulin-like growth factor 2 mRNA-binding protein 1 (IGF2BP1) to EPH receptor A3 (EPHA3) mRNA, thereby increasing EPHA3 expression (76).

In addition to lncRNAs, other classes of ncRNAs also contribute to hepatocarcinogenesis. For instance, circular RNA fatty acid desaturase 1 (circFADS1) has been reported to be upregulated in HCC and is correlated with poor patient outcomes (84). Furthermore, numerous microRNAs (miRNAs) are dysregulated in HCC and regulate key biological processes—including cell proliferation, apoptosis, invasion, and metastasis—by targeting oncogenes or tumor-suppressor genes at the post-transcriptional level (85–87).

### 3.3 Tumor microenvironment

Beyond tumor-intrinsic alterations, the TME represents a critical regulatory layer in HCC pathogenesis. Genetic mutations and epigenetic alterations within tumor cells frequently lead to the activation of oncogenic signaling pathways that reshape the surrounding microenvironment through cytokine secretion and metabolic reprogramming. In turn, stromal and immune cells within the TME further amplify oncogenic signaling and support immune evasion, forming a bidirectional regulatory network that accelerates tumor progression.

#### 3.3.1 Cancer-associated fibroblasts (CAFs)

Cancer-associated fibroblasts (CAFs), the predominant stromal cells in the tumor microenvironment, promote HCC progression mainly through the secretion of cytokines and growth factors that activate oncogenic signaling pathways in tumor cells. These paracrine factors, including TGF-β, interleukin-6 (IL-6), and C-X-C motif chemokine ligand 6 (CXCL6), enhance tumor cell proliferation, stimulate epithelial–mesenchymal transition, and promote angiogenesis while also remodeling the tumor microenvironment and facilitating tumor invasion and metastasis (88,89).

Recent studies have further revealed specific cytokine-mediated signaling networks involved in CAF–tumor cell crosstalk. For instance, CAF-derived cardiotrophin-like cytokine factor 1 (CLCF1) binds to ciliary neurotrophic factor receptors (CNTFRs) on HCC cells and induces the secretion of CXCL6 and TGF-β, forming a cytokine feedback axis that enhances tumor stemness and inflammatory cell recruitment (89). In addition, HCC-derived semaphorin-3C (Sema3C) interacts with neuropilin-1 and integrin β1 on hepatic stellate cells, activating NF-κB signaling and promoting IL-6 release and cholesterol synthesis. CAF-secreted TGF-β1 can further activate AP-1 signaling in tumor cells to upregulate Sema3C expression, thereby establishing a positive feedback loop that accelerates HCC progression (90).

#### 3.3.2 Tumor-associated macrophages (TAMs)

Tumor-associated macrophages (TAMs), an essential component of the tumor microenvironment, can polarize into pro-inflammatory M1 or immunosuppressive M2 phenotypes in response to microenvironmental signals in HCC (91,92). M1 polarization is typically induced by pro-inflammatory stimuli such as interferon-γ (IFN-γ) and lipopolysaccharide (LPS), whereas M2 polarization is promoted by tumor-derived cytokines including interleukin-4 (IL-4), interleukin-10 (IL-10), interleukin-13 (IL-13), and TGF-β (92,93). These signals activate downstream transcriptional programs that drive macrophage polarization and contribute to the establishment of an immunosuppressive tumor microenvironment (91,93).

In HCC, maternal embryonic leucine zipper kinase (MELK) has been reported to interact with and activate signal transducer and activator of transcription 3 (STAT3), thereby promoting STAT3 phosphorylation and enhancing the transcription of its downstream target gene C-C motif chemokine ligand 2 (CCL2) (94). Increased CCL2 expression promotes the recruitment of circulating monocytes and macrophages into the tumor microenvironment and facilitates the accumulation and polarization of TAMs (95,96).

#### 3.3.3 Myeloid-derived suppressor cells (MDSCs)

MDSCs, a heterogeneous population of immunosuppressive myeloid progenitors, expand and activate during tumor progression. They are classified into monocytic (M-MDSCs) and polymorphonuclear (PMN-MDSCs) subsets, with PMN-MDSCs predominating in the HCC microenvironment. Studies have shown that HCC upregulates S100A9 transcription and secretion via the transcription factor ets variant transcription factor 4 (ETV4) (97). The activated MDSCs secrete more S100A9 to activate the ERK/NF-κB signaling pathway in HCC cells, upregulating the expression of ETV5 in a positive feedback loop and forming the “ETV5-S100A9-ERK/NF-κB” self-sustaining circuit (98,99). This dynamically remodels the immunosuppressive microenvironment of HCC, promoting its progression and metastasis.

#### 3.3.4 Immune microenvironment

**FIGURE 3:**
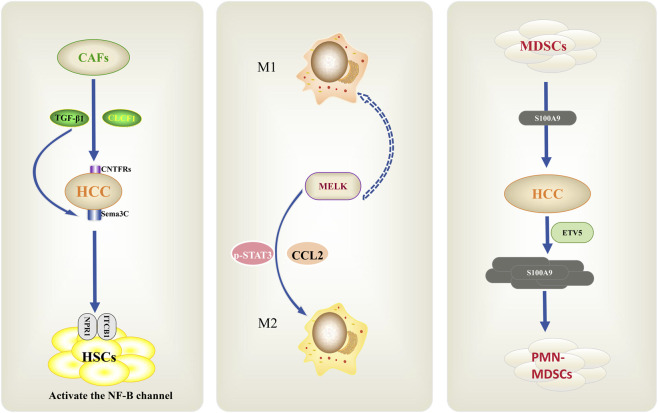
The immunosuppressive tumor microenvironment in HCC. CAFs secrete TGF-β1 and CCL11 to activate HSCs, induce M2 polarization, and recruit MDSCs via NF-κB. They also upregulate MELK/CCL2, boosting MDSC recruitment and M1-to-M2 conversion, thereby establishing an immunosuppressive microenvironment that drives tumor progression.

The immune microenvironment plays a pivotal role in HCC pathogenesis and progression (100). Clinically, high CD8^+^ T-cell infiltration in HCC tissues correlates with favorable prognosis, underscoring their antitumor role. However, the presence of numerous immunosuppressive factors, such as the high expression of the immune checkpoint PD-L1 and cytotoxic T-lymphocyte-associated protein 4 (CTLA-4), can lead to the functional exhaustion of CD8^+^ T cells, causing them to lose their ability to kill tumor cells and create conditions for tumor cell immune escape (101,102). In contrast, regulatory T cells (Tregs) suppress antitumor immunity. Treg levels are elevated in both peripheral blood and tumor tissues of HCC patients, and their infiltration density is associated with advanced tumor stage, vascular invasion, and poor prognosis (Figure 3).

#### 3.3.5 NF-κB-mediated inflammation and immune modulation

The NF-κB pathway serves as a critical molecular link between chronic liver inflammation and hepatocarcinogenesis, functioning primarily within the tumor microenvironment rather than as a cell-autonomous oncogene. More than 80% of HCC cases develop in the context of chronic hepatitis, fibrosis, or cirrhosis, where sustained inflammatory signaling activates NF-κB in both hepatocytes and immune cells (103).

In the tumor microenvironment, NF-κB promotes proliferation and survival by upregulating anti-apoptotic proteins (Bcl-2, Bcl-xL) and cell cycle regulators (Cyclin D1) (104). It functionally interacts with STAT3 and PI3K/AKT pathways, forming a synergistic signaling network that amplifies oncogenic output (105). Notably, NF-κB enhances HCC invasiveness by regulating Snail and Twist, and stimulates angiogenesis through VEGF and IL-8 upregulation (106).

Context-dependent functionality. Paradoxically, in DEN-induced HCC models, NF-κB inhibition promotes tumorigenesis through impaired DNA repair and compensatory STAT3 activation, underscoring its complex role in liver carcinogenesis (107). Clinically, nuclear accumulation of NF-κB correlates with tumor dedifferentiation and advanced TNM stage (108).

### 3.4 Signal transduction pathways

#### 3.4.1 PI3K/AKT/mTOR pathway

**FIGURE 4:**
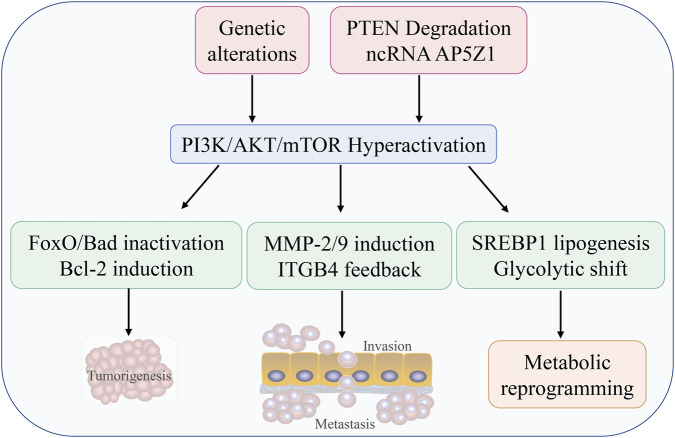
Hyperactivation of PI3K/Akt/mTOR pathway. Genetic alterations activate the PI3K/AKT/mTOR pathway, inactivating FoxO/Bad and inducing Bcl-2 to promote survival, enhancing invasion via MMP-2/9 and ITGB4 feedback, and driving SREBP1-mediated metabolic reprogramming, ultimately leading to tumorigenesis and metastasis.

The PI3K/AKT/mTOR signaling pathway plays a critical role in regulating cell cycle, proliferation, apoptosis, and metabolism in HCC (109–113). Approximately 50% of HCC patients harbor genetic alterations in the pathway, which drives oncogenic signaling through multiple mechanisms (114). Activated AKT phosphorylates and inactivates pro-apoptotic proteins FoxO and Bad, enabling unchecked cell survival (115). This pathway drives metastasis and EMT through MMP-2/9 upregulation and ITGB4. It also orchestrates metabolic reprogramming via SREBP1-dependent lipogenesis and glycolysis, fueling tumor growth (116). Furthermore, the non-coding RNA AP5Z1 and ubiquitination-mediated PTEN degradation exacerbate pathway activation, contributing to HCC progression and therapeutic resistance (117) (Figure 4).

#### 3.4.2 JAK/STAT pathway

The JAK/STAT pathway critically drives HCC progression through constitutive activation, often resulting from STAT3 mutations or JAK2 amplification. This sustained signaling enhances tumor proliferation and therapy resistance by upregulating cyclin D1 and Bcl-2, while inactivating pro-apoptotic proteins FoxO and Bad (118,119). It also interacts with tumor microenvironment components, as STAT3 activation enhances IL-6 secretion, inducing CD8^+^ T cell exhaustion and PD-1/TOX-mediated immunosuppression (120). This pathway also promotes oncogenic amplification through the JAK2/C21orf58/STAT3 ternary complex, where C21orf58 stabilizes STAT3 phosphorylation to confer sorafenib resistance (119).

**FIGURE 5:**
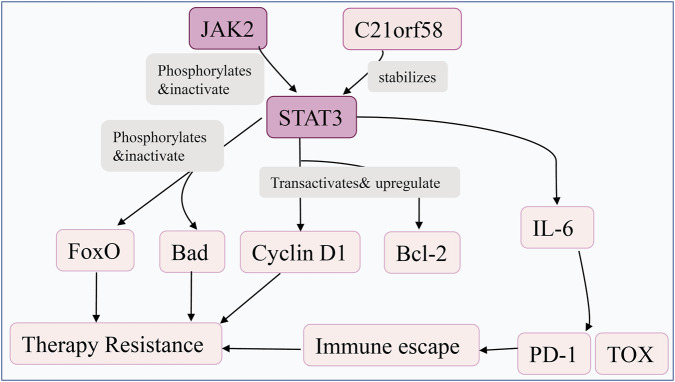
JAK/STAT signaling drives therapy resistance and immune escape. JAK2 phosphorylates and inhibits STAT3, while C21orf58 stabilizes it. STAT3 then activates IL-6, Cyclin D1, and Bcl-2, and inhibits FoxO and Bad, leading to therapy resistance and immune escape via PD-1 and TOX upregulation.

Recent studies further highlight that ncRNAs and epigenetic modifications modulate this axis, with STAT3 activation mutants preferentially binding C21orf58 to amplify oncogenic signals (119), while STAT3 phosphorylation status correlates with poor prognosis, tumor aggressiveness, and recurrence in HCC patients (118) (Figure 5).

#### 3.4.3 TGF-β pathway

**FIGURE 6:**
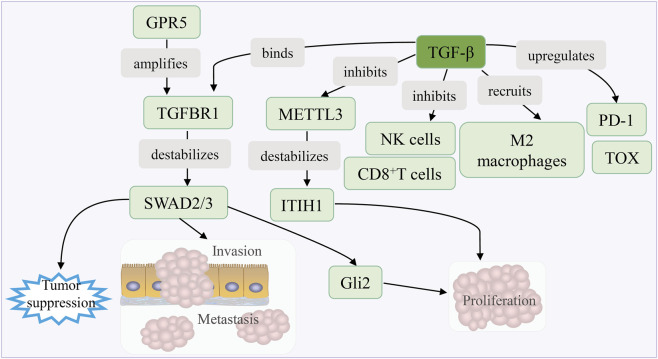
TGF-β axis drives immunosuppression and tumor progression. GPR5 amplification drives invasion by disrupting TGF-β signaling and destabilizing ITIH1. It also suppresses immunity through NK/CD8^+^ T cell inhibition, M2 macrophage recruitment, and PD-1/TOX upregulation. Concurrently, Gli2 promotes proliferation and metastasis, collectively driving tumor progression.

The TGF-β signaling pathway plays a dual and context-dependent role in HCC, acting as a tumor suppressor in the early stages but promoting metastasis and immune evasion in advanced disease. This dichotomy arises from its complex interplay with genetic, epigenetic, and microenvironmental factors (Figure 6).

##### 3.4.3.1 Pro-metastatic and EMT-Promoting effects

In advanced HCC, TGF-β drives EMT and metastasis by activating SMAD2/3-dependent signaling. GPR56, a G protein-coupled receptor overexpressed in HCC, interacts with TGFBR1 to amplify TGF-β signaling, promoting SMAD3 phosphorylation and enhancing EMT markers such as vimentin and claudins while suppressing E-cadherin (121)*.* Additionally, TGF-β enhances integrin-mediated ECM remodeling and MMP activity, further enabling metastatic dissemination (122).

##### 3.4.3.2 Metabolic reprogramming and crosstalk with epigenetic modifications

TGF-β intersects with m6A RNA methylation to regulate HCC progression. It promotes the liquid-liquid phase separation of METTL3, a key m6A methyltransferase, leading to destabilization of ITIH1 mRNA. Loss of ITIH1 due to TGF-β-driven m6A modification accelerates HCC growth and metastasis, whereas the recombinant ITIH1 protein synergizes with TGF-β inhibitors to suppress tumor progression in preclinical models (123).

##### 3.4.3.3 Immune evasion and microenvironment remodeling

TGF-β fosters the TME by recruiting M2-polarized macrophages and Tregs, while directly inhibiting cytotoxic CD8^+^T and NK cell functions. HCC-derived TGF-β suppresses NK cell activity by upregulating PD-1/TOX-mediated exhaustion pathways. Blocking TGF-β signaling in iPSC-derived NK cells restores their antitumor efficacy, particularly when combined with CAR targeting of GPC3 or AFP (124). Furthermore, TGF-β induces ROS/NO production and ATM/ATR/DNA-PK hyperactivation in bystander cells, exacerbating DNA damage and immune dysfunction (125).

##### 3.4.3.4 Dual role in tumor suppression and promotion

In preneoplastic stages, TGF-β suppresses proliferation via SMAD-mediated cell cycle arrest and apoptosis. However, in advanced HCC, *CTNNB1* mutations and dysregulated co-factors like 14-3-3ζ shift the role of TGF-β toward metastasis. 14-3-3ζ destabilizes p53 and stabilizes Gli2, enabling SMAD-Gli2 complexes to drive pro-metastatic genes such as PTHrP, thereby promoting bone metastasis (122).

##### 3.4.3.5 Therapeutic targeting and resistance mechanisms

The combined inhibition of TGF-β and associated pathways shows promise. For example, co-targeting GPR5 and TGFBR1 synergistically reduces metastasis in animal models (121). Similarly, METTL3 inhibitors or m6A modulators can restore ITIH1 expression, counteracting the oncogenic effects of TGF-β (123). However, challenges persist due to pathway redundancy and the dual tumor-suppressive/metastatic roles of TGF-β, necessitating context-specific strategies (122).

#### 3.4.4 Wnt/β-catenin pathway

The Wnt/β-catenin pathway contributes to hepatocellular carcinogenesis through three key mechanisms: genetic alteration, metabolic reprogramming, and crosstalk with other oncogenic cascades. Mutations in CTNNB1 or loss of tumor suppressors such as APC leads to β-catenin nuclear accumulation, which transcriptionally upregulates targets including GLUL (glutamine synthetase, GS) and CCND1, thereby sustaining proliferation and metabolic adaptation (126,127). Elevated GS increases intracellular glutamine, which in turn activates mTORC1 via phosphorylation at Ser2448, promoting cell survival and tumor growth. This “Wnt/β-catenin–GS–glutamine–mTORC1” axis plays a central role in maintaining malignancy in β-catenin-mutated HCC, suggesting mTORC1 inhibition as a potential therapeutic strategy for this subset (127).

**FIGURE 7:**
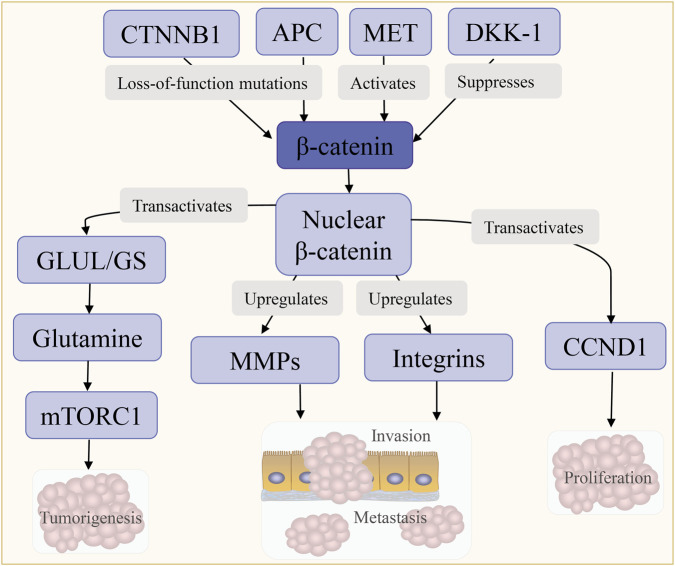
Wnt/β-catenin pathway activation. CTNNB1 loss-of-function mutations relieve β-catenin inhibition by APC, MET, and DKK-1, leading to its nuclear translocation and transactivation of GLUL/GS, MMPs/integrins, CCND1, and mTORC1, ultimately driving tumorigenesis, metastasis, invasion, and proliferation.

Additionally, Wnt/β-catenin pathway further engages in crosstalk with the tumor microenvironment and other oncogenic cascades. Specifically, β-catenin activation promotes EMT and metastatic dissemination through upregulation of MMPs and integrins, which facilitate extracellular matrix degradation and tumor invasion (118,128). Loss-of-function mutations in ion channels like KCNQ1 disrupt epithelial homeostasis by dual mechanisms: by passing FZD/LRP6 to activate MET receptors and suppressing Wnt inhibitor DKK-1, further amplifying β-catenin-driven oncogenesis (129) (Figure 7). Importantly, β-catenin–activated HCC represents a molecular subtype characterized by immune exclusion and limited response to immune checkpoint blockade, highlighting its potential utility as a biomarker for therapeutic stratification (47,48).

#### 3.4.5 Hippo-YAP/TAZ pathway

**FIGURE 8:**
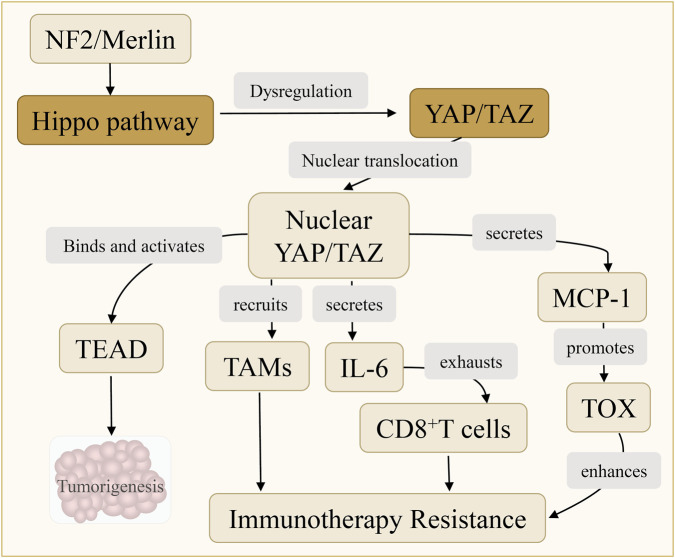
YAP/TAZ activation mediates immunosuppression and therapy resistance. YAP/TAZ activation, triggered by CTNNB1 mutation, epigenetic alterations, or NF2 loss, drives TEAD-mediated transcription, leading to proliferation, metabolic reprogramming, and immune evasion. YAP/TAZ also induce cell competition, recruit TAMs, and secrete IL-6 and MCP-1, promoting CD8^+^ T cell exhaustion and PD-1/TOX-mediated immunotherapy resistance.

The Hippo-YAP/TAZ pathway exerts context-dependent roles in HCC. Nuclear translocation of YAP/TAZ triggered by CTNNB1 mutation, epigenetic alteration, or loss of upstream regulators such as NF2 promotes oncogenic transcription via TEAD, driving proliferation, metabolic reprogramming, and immune evasion. Paradoxically, YAP/TAZ activation in peritumoral hepatocytes suppresses tumor growth through cell competition, inducing apoptosis in adjacent malignant cells and reducing tumor burden by >70% in mice (130). This paradoxical role highlights the spatial and contextual regulation of Hippo signaling. Furthermore, YAP/TAZ enhance immune evasion by recruiting TAMs and secreting the immunosuppressive cytokines IL-6 and MCP-1, which exhaust CD8^+^T cells and promote PD-1/TOX-mediated resistance to immunotherapy (131) (Figure 8).

#### 3.4.6 Convergence of signaling pathways in immune evasion and EMT

Multiple signaling pathways in HCC exhibit functional convergence in promoting immune evasion and EMT, creating redundant mechanisms that sustain tumor progression and therapeutic resistance.

##### 3.4.6.1 Shared immune evasion mechanisms

PI3K/AKT/mTOR, JAK/STAT, and TGF-β pathways collectively contribute to establishing an immunosuppressive microenvironment. Specifically, STAT3 activation (downstream of JAK2 and potentially PI3K) enhances IL-6 secretion, which induces CD8^+^ T cell exhaustion through PD-1/TOX-mediated pathways (120). Similarly, TGF-β signaling directly suppresses cytotoxic CD8^+^ T and NK cell functions, fostering regulatory T cell recruitment (124). These parallel mechanisms suggest that targeting single pathways may be insufficient to reverse immune evasion.

##### 3.4.6.2 EMT and metastatic dissemination

TGF-β represents the primary driver of EMT in advanced HCC, activating SMAD2/3-dependent signaling to enhance vimentin expression while suppressing E-cadherin (121). However, Wnt/β-catenin activation complements this process by upregulating MMPs and integrins, facilitating extracellular matrix degradation (118,128). Additionally, PI3K/AKT/mTOR signaling contributes to metastasis through MMP-2/9 upregulation and ITGB4 modulation (116). The coexistence of these pathways in individual tumors suggests cooperative EMT induction.

##### 3.4.6.3 Metabolic reprogramming interface

These pathways intersect metabolically: TGF-β-driven m6A modification destabilizes ITIH1 mRNA (123), while PI3K/AKT/mTOR activates SREBP1-dependent lipogenesis (116), and Wnt/β-catenin enhances glutamine metabolism via GS-mTORC1 axis (127). This metabolic synergy supports tumor growth under diverse microenvironmental stresses.

##### 3.4.6.4 Therapeutic implications

The extensive crosstalk among these pathways explains the limited efficacy of single-target therapies. For instance, sorafenib resistance frequently involves compensatory activation of JAK/STAT and PI3K/AKT/mTOR pathways. Similarly, β-catenin activation confers resistance to immune checkpoint inhibitors by creating an immune-excluded phenotype. Understanding these convergent nodes may inform rational combination strategies.

#### 3.4.7 Ras/Raf/MEK/ERK pathway

The Ras/Raf/MEK/ERK signaling pathway, a critical branch of the MAPK cascade, plays a central role in HCC pathogenesis through sustained activation of oncogenic signaling, driving uncontrolled proliferation, apoptosis evasion, metastasis, and therapeutic resistance. Its dysregulation in HCC is predominantly triggered by genetic mutations in KRAS, NRAS and BRAF, epigenetic alterations, and aberrant growth factor signaling EGF, VEGF and PDGF (132,133).

**FIGURE 9:**
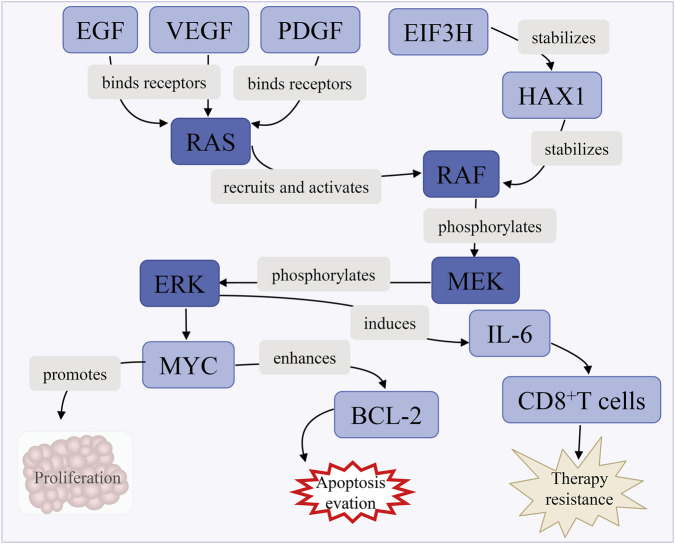
Ras/Raf/MEK/ERK pathway activation. Growth factors (EGF, VEGF, PDGF) activate RAS, triggering the RAF/MEK/ERK cascade. EIF3H and HAX1 enhance signaling by stabilizing RAS and ERK, respectively. Activated ERK induces IL-6 and MYC, with MYC promoting BCL-2, driving proliferation, apoptosis evasion, and therapy resistance.

Activated Ras proteins (KRAS, NRAS, HRAS) recruit the Raf kinase RAF, initiating a phosphorylation cascade that activates MEK and subsequently ERK. Phosphorylated ERK translocates to the nucleus, inducing transcription factors like MYC and FOS, which promote cell cycle progression and suppress apoptosis by inhibiting pro-apoptotic proteins while enhancing anti-apoptotic Bcl-2 expression (133). EIF3H, a eukaryotic translation initiation factor, stabilizes the HAX1 protein to amplify RAF1-MEK-ERK signaling, promoting HCC progression in preclinical models (134). The pathway also interacts with the tumor microenvironment, fostering immune evasion via immunosuppressive cytokine secretion IL-6 and CD8^+^T cell exhaustion (Figure 9).

#### 3.4.8 NRF2/KEAP1

The NRF2/KEAP1 pathway exhibits dual roles in HCC, serving as a cytoprotective mechanism under physiological conditions while promoting oncogenesis upon dysregulation. In normal cells, KEAP1 facilitates NRF2 ubiquitination and proteasomal degradation. In HCC, KEAP1 loss-of-function mutations or other alterations disrupt this interaction, leading to NRF2 stabilization, nuclear accumulation, and constitutive transactivation of antioxidant and detoxification genes. This sustained activation promotes tumor survival, chemoresistance, and metabolic reprogramming, thereby driving HCC progression.

Mutations in KEAP1 or NRF2 are observed in ∼15% of HCC cases, stabilizing NRF2 and amplifying its transcriptional activity (135,136). Additionally, p62 protein aggregates, sequesters KEAP1, prevents NRF2 degradation and perpetuates its activation. MOAP-1, a tumor suppressor, inhibits p62 aggregation and NRF2 hyperactivation by disrupting p62 oligomerization, but its downregulation in HCC exacerbates NRF2-driven oncogenesis (136).

**FIGURE 10:**
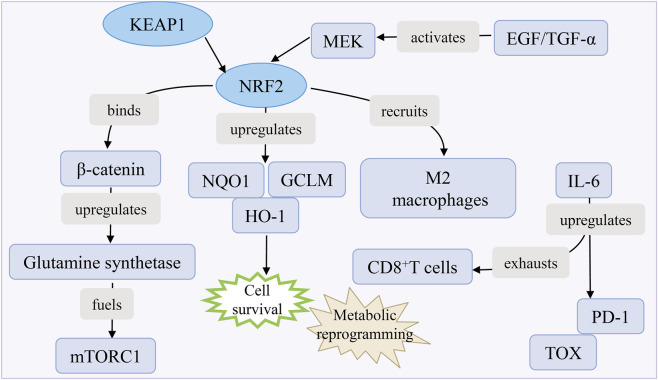
Crosstalk KEAP1/NRF2 pathways in HCC. MEK activates EGF/TGF-α, which drives NRF2-mediated β-catenin upregulation. β-catenin then induces antioxidant genes and glutamine synthetase, fueling mTORC1-driven metabolic reprogramming. This pathway also promotes M2 macrophage polarization and CD8^+^ T cell exhaustion, thereby supporting cell survival.

NRF2 activation upregulates genes like NQO1, HO-1, and GCLM, enhancing glutathione synthesis and detoxification pathways. While this mitigates oxidative stress, it also enables HCC cells to evade ROS-induced apoptosis and resist chemotherapy (135). NRF2 activation is regulated through multiple upstream signals: EGFR ligands such as EGF/TGF-α enhance NRF2 nuclear translocation via MEK-mediated phosphorylation, while cooperation with β-catenin upregulates glutamine synthetase to support mTORC1-driven metabolic adaptation in HCC (135,137). In the tumor microenvironment, NRF2 promotes immunosuppression by inducing M2 macrophage polarization and secreting IL-6, which contributes to CD8^+^ T cell exhaustion and PD-1/TOX upregulation (138) (Figure 10).

#### 3.4.9 Hedgehog pathway

The Hedgehog (Hh) signaling pathway plays a critical oncogenic role in HCC by driving tumor initiation, progression, and therapeutic resistance through genetic dysregulation, stem cell maintenance, metabolic reprogramming, and TME interactions. This process is characterized by the nuclear translocation of Gli transcription factors, which upregulate pro-tumorigenic genes such as CCND1, MYC, and Bcl-2, promoting cell proliferation, apoptosis evasion, and metastasis (139). Gli1 and Gli3 proteins are overexpressed in HCC tissues, with Gli1 expression correlating with tumor dedifferentiation and lymph node metastasis, underscoring their roles as prognostic markers (140,141).

## 4 New advances in the treatment of HCC

### 4.1 Drug therapy

#### 4.1.1 Sorafenib

Sorafenib, the first oral multikinase inhibitor approved for advanced HCC, exerts dual antitumor activity by simultaneously inhibiting vascular endothelial growth factor receptor (VEGFR), platelet-derived growth factor receptor (PDGFR), fibroblast growth factor receptor (FGFR) and key components of the Raf/MEK/ERK pathway. This multi-target inhibition suppresses tumor proliferation and angiogenesis, while additionally inducing ferroptosis through downregulation of glutathione peroxidase glutathione peroxidase 4 (GPX4). With a manageable toxicity profile featuring diarrhea, hand-foot skin reaction, and fatigue, sorafenib remains a standard first-line systemic treatment for surgically unresectable advanced HCC patients with preserved liver function (142–144).

Yu et al. developed an orally administered nanoparticle platform co-loaded with sorafenib and salinomycin for targeted HCC therapy. These butyrate-modified nanoparticles selectively recognize monocarboxylate transporter 1 (MCT-1) on HCC cells, enabling efficient cellular uptake through MCT-1-mediated endocytosis. Upon intracellular release, sorafenib depletes glutathione peroxidase 4 and glutathione, while salinomycin amplifies ferroptosis by elevating intracellular iron levels and lipid peroxidation. This dual-drug combination demonstrated significantly enhanced antitumor efficacy through synergistic induction of ferroptosis and immunogenic cell death (144).

Nevertheless, the clinical efficacy of sorafenib remains limited, demonstrating an objective response rate of merely 2%–3% and a median time to acquired resistance of 6–8 months (145).

#### 4.1.2 Lenvatinib

Lenvatinib, a multi-targeted tyrosine kinase inhibitor, represents a first-line treatment option for advance HCC (146). It exerts antitumor activity by competitively inhibiting kinases including VEGFR, FGFR, KIT, and RET, thereby blocking downstream oncogenic signaling (142). A key mechanism involves the specific suppression of the FGFR4/FGF19 axis, which restores ROS generation and overcomes apoptosis resistance in sorafenib-resistant HCC.

The agent also remodels the tumor immune microenvironment by reducing tumor-associated macrophages, enhancing CD8^+^ T cell infiltration, and activating type I interferon signaling to promote antitumor immunity (147). Furthermore, lenvatinib augments NK cell-mediated cytotoxicity through upregulation of CD16 and NKp46 receptors. However, its efficacy is limited by acquired resistance in approximately 60% of patients within 1 year, and its toxicity profile includes hypertension, proteinuria, and a higher risk of hepatotoxicity compared to sorafenib (147–150).

### 4.2 Immunotherapy

Given the limited surgical eligibility and high recurrence rates in HCC, systemic therapy plays a critical role in disease management. In recent years, immune checkpoint inhibitors (ICIs) has demonstrated remarkable clinical efficacy across diverse solid tumors, including HCC. In addition, there are adoptive cell therapy (ACT) and cancer vaccine strategies, which also show good clinical efficacy (151).

#### 4.2.1 Immune checkpoint inhibitors

In the field of HCC immunotherapy, monotherapy with immune checkpoint inhibitors represented the initial approach in clinical development. Nivolumab, as the first PD-1 inhibitor approved for HCC treatment, marked the beginning of a new era in immunotherapy (152). However, the efficacy of monotherapy remains limited, with objective response rates (ORR) of approximately 15%–20% observed with nivolumab, pembrolizumab, and camrelizumab (153). As monotherapy targets only a single immune checkpoint, it fails to comprehensively reverse the immunosuppressive tumor microenvironment.

To overcome the constraints of monotherapy, combination strategies have become the mainstream approach in HCC immunotherapy. The atezolizumab combined with bevacizumab regimen, significantly improved overall survival and progression-free survival compared with sorafenib, while achieving an objective response rate exceeding 30% (154). Other anti-angiogenic drugs, such as lenvatinib, in combination with immune checkpoint inhibitors, have also shown promising efficacy and safety across multiple clinical studies (155).

#### 4.2.2 Adoptive cell therapy

ACT as an emerging cancer treatment approach has gradually gained prominence in the field of HCC. It involves collecting the patient’s own or donor’s immune cells, expanding, activating or genetically engineering them *in vitro*, and then reinfusing them into the patient to enhance the body’s immune killing ability against tumor cells.

Chimeric antigen receptor T-cell (CAR-T) therapy has shown remarkable success in treating hematological malignancies, but it encounters significant challenges when addressing solid tumors like HCC (156). Tumor cells employ various immune escape mechanisms, and the immunosuppressive characteristics of the HCC tumor microenvironment hinder CAR-T cell infiltration into the cancer tissue. Currently, CAR-T therapy for HCC remains in early developmental phases, with ongoing research focused on optimizing CAR designs through selection of more specific tumor-associated antigens and developing combination approaches to address these limitations (157,158).

T-cell receptor-engineered T-cell (TCR-T) therapy represents another innovative approach under investigation for HCC. By recognizing tumor-associated or tumor-specific antigens presented by MHC molecules, TCR-T cells achieve precise targeting of malignant cells (159).

#### 4.2.3 Cancer vaccine strategies

Cancer vaccines are broadly categorized as preventive or therapeutic (160), Various vaccine platforms are under investigation for HCC. Peptide-based vaccines utilize tumor-associated antigen epitopes to stimulate immune responses [152], yet the identification of universally effective peptides remains challenging due to high tumor heterogeneity in HCC (161). Dendritic cell (DC) vaccines have demonstrated potential in enhancing antitumor immunity, though their complex manufacturing process and high costs impede broad clinical application (162).

Despite advances in HCC vaccine development, several challenges persist. Tumor heterogeneity impedes the creation of a universally effective vaccine, furthermore, the immunosuppressive tumor microenvironment in HCC can neutralize vaccine-induced immune responses, undermining therapeutic effectiveness (163).

### 4.3 Arginine deprivation therapy

HCC exhibits increased reliance on arginine due to deficient expression of arginine-regenerating enzymes ASS and OTC. Preclinical studies indicate that arginine-degrading enzymes such as arginine deiminase (ADI) demonstrate efficacy in ASS-deficient tumors. The pegylated form ADI-PEG 20 has been applied in clinical settings for HCC patients with low or undetectable ASS activity. Although absolute ASS levels in HCC tissues appear relatively high, argininosuccinate synthase 1 (ASS1) expression remains substantially lower than in normal liver tissue. Notably, preclinical evidence suggests that HCC cases with reduced ASS expression show heightened sensitivity to arginine deprivation therapy (164).

A clinical trial involving seven patients with locally advanced or metastatic HCC demonstrated consistent tumor regression following arginine deprivation, indicating its systemic antitumor efficacy against HCC (165). However, the efficacy of arginine deprivation therapy in HCC is variable. Resistance to ADI treatment occurs in some ASS-positive HCC cell lines, while clinical application faces challenges including tumor heterogeneity, potential ASS1 re-expression, and modulation by the tumor microenvironment. These factors contribute to inconsistent treatment responses, hindering broad clinical adoption of this approach.

### 4.4 RNA nanotherapy

RNA nanotherapeutics can directly regulate gene expression associated with tumor progression, and are effectively protected and delivered to the tumor site by nanocarriers such as lipid nanoparticles (LNPs), polymer nanoparticles (PNPs) and bioengineered carriers (166–170). By functionalizing tumor-specific ligands or antibodies on the surface of the nanoparticles, the nanoparticles can selectively bind to receptors that are overexpressed by HCC cells, such as Glypican-3 or transferrin receptors (171,172).

Liu et al, assembled innovative GNPs for hPD-L1 siRNA delivery, which in combination with photothermal therapy significantly inhibited HCC cell growth and downregulated hPD-L1 expression. Nanotechnology combined with photothermal therapy is the activation of nanomaterials by near-infrared light irradiation to generate heat or reactive oxygen species for the precise treatment of HCC (173). Li et al, developed lactic acid (LA)-modified redox-responsive nanoparticles (LA NPs) for the delivery of 10-hydroxycamptothecin poly and sibcl-2 RNA for synergistic anti-HCC. The overexpression of the ASGP receptor was specifically recognized by LA (174). The first saRNA drug to enter clinical trials, MTL-CEBPA (NCT02716012), has shown promising safety and therapeutic promise when used in combination with TKIs, and may become the first RNA drug for HCC therapy (175).

### 4.5 Artificial intelligence (AI)

**FIGURE 11:**
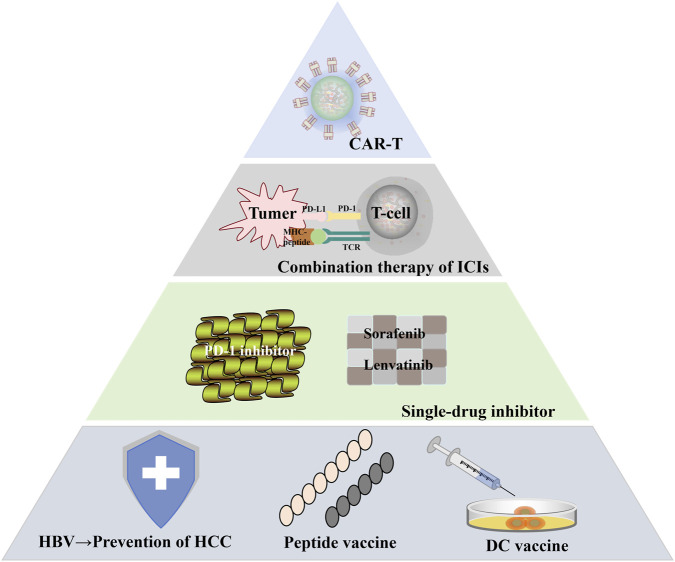
Current and emerging therapeutic strategies for HCC.

In terms of diagnosis, AI technology plays a significant role in non-invasive imaging modes such as ultrasound, CT and MRI, helping to detect and classify liver nodules and liver fibrosis. In terms of liver nodule detection, algorithms based on deep learning can efficiently identify tiny lesions and distinguish between benign and malignant lesions. Its diagnostic accuracy rate has approached or even surpassed that of experienced radiologists (176,177). Furthermore, AI is also used in liquid biopsy, combined with artificial neural networks and decision tree data mining methods, to analyze biomarkers such as heat shock 70 kDa protein 8 isoform type 2, cytochrome b5 and cathepsin B, in order to identify biomarkers of HCC (178,179). In terms of treatment, AI also shows great potential. The application of surgical robots in liver tumor resection can enhance surgical efficiency and improve the postoperative quality of life for patients (180–183). The recent advances in HCC treatment are summarized in Figure 11.

## 5 Discussion

HCC remains a major global health challenge due to its high molecular heterogeneity and diagnostic complexities. This review summarizes recent advances in HCC pathogenesis and emerging therapeutic strategies (Table 5), and discusses key mechanistic insights as well as remaining clinical challenges.

**TABLE 5:** Molecular alterations and corresponding therapeutic strategies in HCC.

Molecular alteration	Biogical impact	Therapeutic approach
TERT promoter mutation	Telomerase reactivation; genomic instability; hepatocyte immortalization	Telomerase inhibitors; Wnt/β-catenin pathway inhibitors
TP53 mutation (R249S)	Aggressive tumor behavior; high recurrence rates and poor prognosis; Flatoxin B1 and HBV infection	MDM2 inhibitors (investigational)
CTNNB1 mutation (∼18.5%) and AXIN1 mutation (∼8%)	Wnt/β-catenin pathway activation; metabolic reprogramming; reduced responsiveness to ICIs	mTORC1 inhibition; β-catenin inhibitors; combination with telomerase inhibitors
KEAP1/NRF2 mutation (∼15%)	NRF2 activation enhanced antioxidant capacity; M2 macrophage polarization CD8^+^ T cell exhaustion	NRF2 inhibitors; glutaminase inhibitors; combination with immunotherapy
ARID1A/ARID2 mutation	Chromatin remodeling; DNA repair deficiency	Synthetic lethal strategies; PARP inhibitors; EZH2 inhibitors
BRD7 truncation	Loss of tumor suppressor function	BRD7 restoration strategies
RB1 pathway alteration	PI3K pathway dysregulation; disruption of G1/S cell cycle checkpoint; proliferation; cell cycle dysregulation	CDK4/6 inhibitors; CDK inhibitors
PI3K/AKT/mTOR pathway activation (∼50%)	Cell survival, proliferation, metabolism, apoptosis evasion, metastasis	mTOR inhibitors; AKT inhibitors; combination with immunotherapy
CDKN2A mutation	Loss of p16INK4a function; metabolic reprogramming	With RB1 pathway targeting; PI3K inhibitors
JAK/STAT pathway activation	Constitutive STAT3 phosphorylation; IL-6-mediated CD8^+^ T cell exhaustion; CCL2-dependent immunosuppression	JAK2 inhibitors; STAT3 inhibitors; combination with CCR2 antagonists
TGF-β pathway activation	SMAD2/3-dependent metastasis; immune evasion; metabolic reprogramming	TGF-β inhibitor; combination with anti-angiogenics
Wnt/β-catenin pathway activation	Glutamine metabolism activation; EMT, MMP upregulation; immune exclusion	Glutamine synthetase inhibitors; mTORC1 inhibition; EMT inhibitors
Hippo-YAP/TAZ activation	TEAD-mediated proliferation; tumor suppression; immunosuppressive cytokine secretion	YAP/TAZ inhibitors; TEAD inhibitors
Ras/Raf/MEK/ERK pathway activation	Apoptosis evasion; metastasis; immunosuppressive cytokine secretion	Sorafenib (multi-kinase inhibitor); MEK inhibitors; combination with immunotherapy

HCC arises from the coordinated interplay of genetic mutations, epigenetic dysregulation, oncogenic signaling pathways, and tumor microenvironmental remodeling, which together form an interconnected regulatory network. In this framework, genetic alterations act as the initiating drivers, epigenetic mechanisms reshape transcriptional landscapes, and oncogenic signaling cascades integrate these molecular inputs to regulate tumor cell proliferation, metabolism, and survival. Meanwhile, interactions with the tumor microenvironment—including immunosuppressive cells such as tumor-associated macrophages, regulatory T cells, and myeloid-derived suppressor cells—create a permissive niche that further reinforces these oncogenic processes. This integrated perspective highlights the multi-layered nature of hepatocarcinogenesis and provides a conceptual basis for the development of combination therapeutic strategies. Recurrent genetic events are key drivers of hepatocarcinogenesis. These include mutations in TERT, TP53, and the Wnt/β-catenin pathway that promote genomic instability, metabolic reprogramming, and uncontrolled proliferation. TERT promoter mutations frequently co-occur with β-catenin activation. In parallel, epigenetic dysregulation contributes to malignant transformation by facilitating oncogene activation and silencing tumor-suppressor genes. Non-coding RNAs further modulate these processes through post-transcriptional regulation, amplifying tumor growth, invasion, and metastatic potential. Beyond tumor-intrinsic alterations, the TME plays a critical role in shaping disease progression. Immunosuppressive components such as tumor-associated macrophages, regulatory T cells, and myeloid-derived suppressor cells establish a permissive niche that supports immune evasion and therapeutic resistance. Moreover, signaling pathways such as Hippo-YAP/TAZ exhibit context-dependent functions in HCC, reflecting the complex regulatory networks that sustain tumor development.

Despite significant advances in systemic therapy, several challenges limit the long-term clinical management of HCC. One major obstacle is the development of therapeutic resistance. Targeted therapies, including sorafenib and lenvatinib, have improved survival in patients with advanced disease; however, both primary and acquired resistance frequently occur. Mechanistically, resistance is often associated with the activation of compensatory pathways such as PI3K/AKT/mTOR, STAT3, and alternative angiogenic signaling through the FGFR4/FGF19 axis. Additionally, metabolic reprogramming, epigenetic alterations, and dynamic interactions within the TME can further reduce drug sensitivity and promote tumor survival, ultimately limiting the durability of targeted therapies.

Another important limitation is the lack of reliable predictive biomarkers for treatment response. Although immune checkpoint inhibitors have transformed the therapeutic landscape of HCC, only a subset of patients achieve durable clinical benefit. Several candidate biomarkers have been proposed, including PD-L1 expression, tumor mutational burden, immune cell infiltration patterns, and activation of the Wnt/β-catenin pathway. Notably, tumors with β-catenin activation frequently exhibit an immune-excluded phenotype and are often less responsive to immunotherapy. In addition, metabolic factors such as ASS1 expression may influence the efficacy of arginine deprivation strategies, highlighting the importance of metabolic heterogeneity in therapeutic response.

Importantly, these molecular alterations not only drive tumor development but also provide clinically relevant biomarkers that may guide prognosis assessment, patient stratification, and personalized therapeutic strategies. Emerging therapeutic approaches aim to overcome these limitations by targeting multiple hallmarks of HCC simultaneously. Combination strategies integrating targeted therapy and immunotherapy have demonstrated improved clinical outcomes compared with monotherapy. In parallel, novel technologies—including nanoparticle-based drug delivery systems and RNA-based therapeutics—are being explored to enhance drug delivery, induce immunogenic cell death, and modulate tumor metabolism. Advances in AI also offer promising opportunities for improving early detection and treatment monitoring through imaging analysis and liquid biopsy-based biomarker identification. However, these approaches still require validation in large prospective cohorts.

Looking forward, the successful translation of mechanistic insights into clinical benefit will depend on the development of precision medicine strategies. Future efforts should prioritize the identification of robust combinatorial biomarkers for patient stratification and therapeutic decision-making. In addition, integrating multi-omics profiling with longitudinal monitoring approaches, such as liquid biopsy and advanced imaging analytics, may enable dynamic assessment of tumor evolution and treatment response. Importantly, translating these discoveries into clinical practice will require well-designed prospective trials and interdisciplinary collaboration between basic researchers, clinicians, and data scientists. Such integrative strategies may ultimately facilitate personalized therapeutic regimens that simultaneously target oncogenic signaling pathways and the immunosuppressive tumor microenvironment, thereby improving long-term clinical outcomes for patients with hepatocellular carcinoma.

## References

[1] Forner A Reig M Bruix J . Hepatocellular Carcinoma. Lancet (2018) 391(10127):1301–14. 10.1016/S0140-6736(18)30010-2 29307467

[2] Nagaraju GP Dariya B Kasa P Peela S El-Rayes BF . Epigenetics in hepatocellular carcinoma. Semin Cancer Biol (2022) 86(Pt 3):622–32. 10.1016/j.semcancer.2021.07.017 34324953

[3] Villanueva A . Hepatocellular carcinoma. N Engl J Med (2019) 380(15):1450–62. 10.1056/NEJMra1713263 30970190

[4] Sung H Ferlay J Siegel RL Laversanne M Soerjomataram I Jemal A Global cancer statistics 2020: GLOBOCAN estimates of incidence and mortality worldwide for 36 cancers in 185 countries. CA Cancer J Clin (2021) 71(3):209–49. 10.3322/caac.21660 33538338

[5] European Association For The Study Of The L, European Organisation For RTreatment Of C. EASL-EORTC clinical practice guidelines: management of hepatocellular carcinoma. J Hepatol (2012) 56(4):908–43. 10.1016/j.jhep.2011.12.001 22424438

[6] El-Serag HB Rudolph KL . Hepatocellular carcinoma: epidemiology and molecular carcinogenesis. Gastroenterology (2007) 132(7):2557–76. 10.1053/j.gastro.2007.04.061 17570226

[7] Allemani C Weir HK Carreira H Harewood R Spika D Wang XS Global surveillance of cancer survival 1995-2009: analysis of individual data for 25,676,887 patients from 279 population-based registries in 67 countries (CONCORD-2). Lancet (2015) 385(9972):977–1010. 10.1016/S0140-6736(14)62038-9 25467588 PMC4588097

[8] Serper M Taddei TH Mehta R D'Addeo K Dai F Aytaman A Association of provider specialty and multidisciplinary care with hepatocellular carcinoma treatment and mortality. Gastroenterology (2017) 152(8):1954–64. 10.1053/j.gastro.2017.02.040 28283421 PMC5664153

[9] Delire B Starkel P . The Ras/MAPK pathway and hepatocarcinoma: pathogenesis and therapeutic implications. Eur J Clin Invest (2015) 45(6):609–23. 10.1111/eci.12441 25832714

[10] der Stroth L Tharehalli U Gunes C Lechel A , editors Telomeres and telomerase in the development of liver cancer, Cancers (Basel) (2020) 12(8):2048. 10.3390/cancers12082048 32722302 PMC7464754

[11] Roxburgh P Evans TR . Systemic therapy of hepatocellular carcinoma: are we making progress? Adv Ther (2008) 25(11):1089–104. 10.1007/s12325-008-0113-z 18972075

[12] Foglia B Turato C Cannito S . Hepatocellular carcinoma: latest research in pathogenesis, detection and treatment. Int J Mol Sci (2023) 24(15):12224. 10.3390/ijms241512224 37569600 PMC10419038

[13] Bianco C Jamialahmadi O Pelusi S Baselli G Dongiovanni P Zanoni I Non-invasive stratification of hepatocellular carcinoma risk in non-alcoholic fatty liver using polygenic risk scores. J Hepatol (2021) 74(4):775–82. 10.1016/j.jhep.2020.11.024 33248170 PMC7987554

[14] Toh MR Wong EYT Wong SH Ng AWT Loo LH Chow PK Global epidemiology and genetics of hepatocellular carcinoma. Gastroenterology (2023) 164(5):766–82. 10.1053/j.gastro.2023.01.033 36738977

[15] Atkins JL Pilling LC Masoli JAH Kuo CL Shearman JD Adams PC Association of hemochromatosis HFE p.C282Y homozygosity with hepatic malignancy. JAMA (2020) 324(20):2048–57. 10.1001/jama.2020.21566 33231665 PMC7686863

[16] Stewart MF . Review of hepatocellular cancer, hypertension and renal impairment as late complications of acute porphyria and recommendations for patient follow-up. J Clin Pathol (2012) 65(11):976–80. 10.1136/jclinpath-2012-200791 22851509

[17] Linet MS Gridley G Nyren O Mellemkjaer L Olsen JH Keehn S Primary liver cancer, other malignancies, and mortality risks following porphyria: a cohort study in Denmark and Sweden. Am J Epidemiol (1999) 149(11):1010–5. 10.1093/oxfordjournals.aje.a009745 10355376

[18] Whitfield JB Schwantes-An TH Darlay R Aithal GP Atkinson SR Bataller R A genetic risk score and diabetes predict development of alcohol-related cirrhosis in drinkers. J Hepatol (2022) 76(2):275–82. 10.1016/j.jhep.2021.10.005 34656649 PMC8803006

[19] Huang C Li Y Zhang F Zhang C Ding Z . Advancements in elucidating the mechanisms of Sorafenib resistance in hepatocellular carcinoma. Int J Surg (2025) 111(4):2990–3005. 10.1097/JS9.0000000000002294 39992113 PMC12175829

[20] Brown ZJ Tsilimigras DI Ruff SM Mohseni A Kamel IR Cloyd JM Management of hepatocellular carcinoma: a review. JAMA Surg (2023) 158(4):410–20. 10.1001/jamasurg.2022.7989 36790767

[21] Bruix J Boix L Sala M Llovet JM . Focus on hepatocellular carcinoma. Cancer Cell (2004) 5(3):215–9. 10.1016/s1535-6108(04)00058-3 15050913

[22] Jihye C Jinsil S . Application of radiotherapeutic strategies in the BCLC-Defined stages of hepatocellular carcinoma. Liver Cancer (2012) 1(3-4):216–25. 10.1159/000343836 24159586 PMC3760456

[23] Huang J Osarogiagbon RU Giroux DJ Nishimura KK Bille A Cardillo G The International association for the study of lung cancer staging project for lung cancer: proposals for the revision of the N descriptors in the forthcoming ninth edition of the TNM classification for lung cancer. J Thorac Oncol (2024) 19(5):766–85. 10.1016/j.jtho.2023.10.012 37866624 PMC12323887

[24] Rami-Porta R Nishimura KK Giroux DJ Detterbeck F Cardillo G Edwards JG The international Association for the Study of lung cancer lung cancer staging Project: proposals for revision of the TNM stage groups in the forthcoming (ninth) edition of the TNM classification for lung cancer. J Thorac Oncol (2024) 19(7):1007–27. 10.1016/j.jtho.2024.02.011 38447919

[25] Okuda K Ohtsuki T Obata H Tomimatsu M Okazaki N Hasegawa H Natural history of hepatocellular carcinoma and prognosis in relation to treatment. Study of 850 patients. Cancer. (1985) 56(4):918–28. 10.1002/1097-0142(19850815)56:4<918::aid-cncr2820560437>3.0.co;2-e 2990661

[26] Ferlicot S Paradis V Dargère D Monges G Bedossa P . Detection of telomerase in hepatocellular carcinomas using a PCR ELISA assay: comparison with hTR expression. J Clin Pathol (1999) 52(10):725–9. 10.1136/jcp.52.10.725 10674027 PMC501563

[27] Nakayama J Tahara H Tahara E Saito M Ito K Nakamura H Telomerase activation by hTRT in human normal fibroblasts and hepatocellular carcinomas. Nat Genet (1998) 18(1):65–8. 10.1038/ng0198-65 9425903

[28] Shimada M Hasegawa H Gion T Utsunomiya T Shirabe K Takenaka K The role of telomerase activity in hepatocellular carcinoma. Am J Gastroenterol (2000) 95(3):748–52. 10.1111/j.1572-0241.2000.01855.x 10710069

[29] Nault JC Ningarhari M Rebouissou S Zucman-Rossi J . The role of telomeres and telomerase in cirrhosis and liver cancer. Nat Rev Gastroenterol Hepatol (2019) 16(9):544–58. 10.1038/s41575-019-0165-3 31253940

[30] Bayard Q Meunier L Peneau C Renault V Shinde J Nault JC Cyclin A2/E1 activation defines a hepatocellular carcinoma subclass with a rearrangement signature of replication stress. Nat Commun (2018) 9(1):5235. 10.1038/s41467-018-07552-9 30531861 PMC6286353

[31] Logan GJ Dane AP Hallwirth CV Smyth CM Wilkie EE Amaya AK Identification of liver-specific enhancer-promoter activity in the 3' untranslated region of the wild-type AAV2 genome. Nat Genet (2017) 49(8):1267–73. 10.1038/ng.3893 28628105

[32] Nault JC Mallet M Pilati C Calderaro J Bioulac-Sage P Laurent C High frequency of telomerase reverse-transcriptase promoter somatic mutations in hepatocellular carcinoma and preneoplastic lesions. Nat Commun (2013) 4:2218. 10.1038/ncomms3218 23887712 PMC3731665

[33] Sung WK Zheng H Li S Chen R Liu X Li Y Genome-wide survey of recurrent HBV integration in hepatocellular carcinoma. Nat Genet (2012) 44(7):765–9. 10.1038/ng.2295 22634754

[34] Totoki Y Tatsuno K Covington KR Ueda H Creighton CJ Kato M Trans-ancestry mutational landscape of hepatocellular carcinoma genomes. Nat Genet (2014) 46(12):1267–73. 10.1038/ng.3126 25362482

[35] Cesare AJ Reddel RR . Alternative lengthening of telomeres: models, mechanisms and implications. Nat Rev Genet (2010) 11(5):319–30. 10.1038/nrg2763 20351727

[36] Wood LD Heaphy CM Daniel HD Naini BV Lassman CR Arroyo MR Chromophobe hepatocellular carcinoma with abrupt anaplasia: a proposal for a new subtype of hepatocellular carcinoma with unique morphological and molecular features. Mod Pathol (2013) 26(12):1586–93. 10.1038/modpathol.2013.68 23640129 PMC3974906

[37] Kim E Viatour P . Hepatocellular carcinoma: old friends and new tricks. Exp Mol Med (2020) 52(12):1898–907. 10.1038/s12276-020-00527-1 33268834 PMC8080814

[38] Bressac B Kew M Wands J Ozturk M . Selective G to T mutations of p53 gene in hepatocellular carcinoma from southern Africa. Nature (1991) 350(6317):429–31. 10.1038/350429a0 1672732

[39] Levrero M Zucman-Rossi J . Mechanisms of HBV-induced hepatocellular carcinoma. J Hepatol (2016) 64(1 Suppl. l):S84–s101. 10.1016/j.jhep.2016.02.021 27084040

[40] Comprehensive and integrative genomic characterization of hepatocellular carcinoma. Cell. (2017) 169(7):1327–41.e23. 10.1016/j.cell.2017.05.046 28622513 PMC5680778

[41] Nault JC Villanueva A . Biomarkers for hepatobiliary cancers. Hepatology (2021) 73(Suppl. 1):115–27. 10.1002/hep.31175 32045030

[42] Schulze K Imbeaud S Letouzé E Alexandrov LB Calderaro J Rebouissou S Exome sequencing of hepatocellular carcinomas identifies new mutational signatures and potential therapeutic targets. Nat Genet (2015) 47(5):505–11. 10.1038/ng.3252 25822088 PMC4587544

[43] Park JI Venteicher AS Hong JY Choi J Jun S Shkreli M Telomerase modulates Wnt signalling by association with target gene chromatin. Nature (2009) 460(7251):66–72. 10.1038/nature08137 19571879 PMC4349391

[44] Rebouissou S Franconi A Calderaro J Letouzé E Imbeaud S Pilati C Genotype-phenotype correlation of CTNNB1 mutations reveals different ß-catenin activity associated with liver tumor progression. Hepatology (2016) 64(6):2047–61. 10.1002/hep.28638 27177928

[45] Perugorria MJ Olaizola P Labiano I Esparza-Baquer A Marzioni M Marin JJG Wnt-β-catenin signalling in liver development, health and disease. Nat Rev Gastroenterol Hepatol (2019) 16(2):121–36. 10.1038/s41575-018-0075-9 30451972

[46] Colnot S Decaens T Niwa-Kawakita M Godard C Hamard G Kahn A Liver-targeted disruption of Apc in mice activates beta-catenin signaling and leads to hepatocellular carcinomas. Proc Natl Acad Sci U S A. (2004) 101(49):17216–21. 10.1073/pnas.0404761101 15563600 PMC535370

[47] Harding JJ Nandakumar S Armenia J Khalil DN Albano M Ly M Prospective genotyping of hepatocellular carcinoma: clinical implications of next-generation sequencing for matching patients to targeted and immune therapies. Clin Cancer Res (2019) 25(7):2116–26. 10.1158/1078-0432.CCR-18-2293 30373752 PMC6689131

[48] Ruiz de Galarreta M Bresnahan E Molina-Sánchez P Lindblad KE Maier B Sia D β-Catenin activation promotes immune escape and resistance to Anti-PD-1 therapy in hepatocellular carcinoma. Cancer Discov (2019) 9(8):1124–41. 10.1158/2159-8290.CD-19-0074 31186238 PMC6677618

[49] Bouattour M Mehta N He AR Cohen EI Nault JC . Systemic treatment for advanced hepatocellular carcinoma. Liver Cancer (2019) 8(5):341–58. 10.1159/000496439 31768344 PMC6873089

[50] Li M Zhao H Zhang X Wood LD Anders RA Choti MA Inactivating mutations of the chromatin remodeling gene ARID2 in hepatocellular carcinoma. Nat Genet (2011) 43(9):828–9. 10.1038/ng.903 21822264 PMC3163746

[51] Guichard C Amaddeo G Imbeaud S Ladeiro Y Pelletier L Maad IB Integrated analysis of somatic mutations and focal copy-number changes identifies key genes and pathways in hepatocellular carcinoma. Nat Genet (2012) 44(6):694–8. 10.1038/ng.2256 22561517 PMC3819251

[52] Huang W Skanderup AJ Lee CG . Advances in genomic hepatocellular carcinoma research. Gigascience (2018) 7(11):giy135. 10.1093/gigascience/giy135 30521023 PMC6335342

[53] Tong S Revill P . Overview of hepatitis B viral replication and genetic variability. J Hepatol (2016) 64(1 Suppl. l):S4–s16. 10.1016/j.jhep.2016.01.027 27084035 PMC4834849

[54] Chen CL Wang Y Pan QZ Tang Y Wang QJ Pan K Bromodomain-containing protein 7 (BRD7) as a potential tumor suppressor in hepatocellular carcinoma. Oncotarget (2016) 7(13):16248–61. 10.18632/oncotarget.7637 26919247 PMC4941311

[55] Chiu YH Lee JY Cantley LC . BRD7, a tumor suppressor, interacts with p85α and regulates PI3K activity. Mol Cell (2014) 54(1):193–202. 10.1016/j.molcel.2014.02.016 24657164 PMC4004185

[56] Fujimoto A Furuta M Totoki Y Tsunoda T Kato M Shiraishi Y Whole-genome mutational landscape and characterization of noncoding and structural mutations in liver cancer. Nat Genet (2016) 48(5):500–9. 10.1038/ng.3547 27064257

[57] Zhang Q Wei L Yang H Yang W Yang Q Zhang Z Bromodomain containing protein represses the Ras/Raf/MEK/ERK pathway to attenuate human hepatoma cell proliferation during HCV infection. Cancer Lett (2016) 371(1):107–16. 10.1016/j.canlet.2015.11.027 26620707

[58] Dyson NJ . RB1: a prototype tumor suppressor and an enigma. Genes Dev (2016) 30(13):1492–502. 10.1101/gad.282145.116 27401552 PMC4949322

[59] Kent LN Leone G . The broken cycle: E2F dysfunction in cancer. Nat Rev Cancer (2019) 19(6):326–38. 10.1038/s41568-019-0143-7 31053804

[60] Knudsen ES Knudsen KE . Tailoring to RB: tumour suppressor status and therapeutic response. Nat Rev Cancer (2008) 8(9):714–24. 10.1038/nrc2401 19143056 PMC2914856

[61] Rao S Lee SY Gutierrez A Perrigoue J Thapa RJ Tu Z Inactivation of ribosomal protein L22 promotes transformation by induction of the stemness factor, Lin28B. Blood (2012) 120(18):3764–73. 10.1182/blood-2012-03-415349 22976955 PMC3488889

[62] Weinstein HNW Hu K Fish L Chen YA Allegakoen P Pham JH RPL22 is a tumor suppressor in MSI-high cancers and a splicing regulator of MDM4. Cell Rep (2024) 43(8):114622. 10.1016/j.celrep.2024.114622 39146182 PMC12035866

[63] Lu SC Mato JM . S-adenosylmethionine in liver health, injury, and cancer. Physiol Rev (2012) 92(4):1515–42. 10.1152/physrev.00047.2011 23073625 PMC3698976

[64] Park S Hall MN . Metabolic reprogramming in hepatocellular carcinoma: mechanisms and therapeutic implications. Exp Mol Med (2025) 57(3):515–23. 10.1038/s12276-025-01415-2 40025169 PMC11958682

[65] Zucman-Rossi J Villanueva A Nault JC Llovet JM . Genetic landscape and biomarkers of hepatocellular carcinoma. Gastroenterology (2015) 149(5):1226–39.e4. 10.1053/j.gastro.2015.05.061 26099527

[66] Zhou L Yu CW . Epigenetic modulations in triple-negative breast cancer: therapeutic implications for tumor microenvironment. Pharmacol Res (2024) 204:107205. 10.1016/j.phrs.2024.107205 38719195

[67] Wang N Ma T Yu B . Targeting epigenetic regulators to overcome drug resistance in cancers. Signal Transduct Target Ther (2023) 8(1):69. 10.1038/s41392-023-01341-7 36797239 PMC9935618

[68] Baylin SB Jones PA . A decade of exploring the cancer epigenome - biological and translational implications. Nat Rev Cancer (2011) 11(10):726–34. 10.1038/nrc3130 21941284 PMC3307543

[69] Kong LM Liao CG Chen L Yang HS Zhang SH Zhang Z Promoter hypomethylation up-regulates CD147 expression through increasing Sp1 binding and associates with poor prognosis in human hepatocellular carcinoma. J Cell Mol Med (2011) 15(6):1415–28. 10.1111/j.1582-4934.2010.01124.x 20629990 PMC4373337

[70] Liao CG Liang XH Ke Y Yao L Liu M Liu ZK Active demethylation upregulates CD147 expression promoting non-small cell lung cancer invasion and metastasis. Oncogene (2022) 41(12):1780–94. 10.1038/s41388-022-02213-0 35132181 PMC8933279

[71] Wang S-J Chao D Wei W Nan G Li J-Y Liu F-L CD147 promotes collective invasion through cathepsin B in hepatocellular carcinoma. J Exp & Clin Cancer Res (2020) 39(1):145. 10.1186/s13046-020-01647-2 32727598 PMC7391525

[72] Xiong L Wu F Wu Q Xu L Cheung OK Kang W Aberrant enhancer hypomethylation contributes to hepatic carcinogenesis through global transcriptional reprogramming. Nat Commun (2019) 10(1):335. 10.1038/s41467-018-08245-z 30659195 PMC6338783

[73] Zhou Q Yin Y Yu M Gao D Sun J Yang Z GTPBP4 promotes hepatocellular carcinoma progression and metastasis *via* the PKM2 dependent glucose metabolism. Redox Biol (2022) 56:102458. 10.1016/j.redox.2022.102458 36116159 PMC9483790

[74] Zhang J Zhang H Ding X Hu J Li Y Zhang J Crosstalk between macrophage-derived PGE(2) and tumor UHRF1 drives hepatocellular carcinoma progression. Theranostics (2022) 12(8):3776–93. 10.7150/thno.69494 35664070 PMC9131282

[75] Zhang R Dai J Yao F Zhou S Huang W Xu J Hypomethylation-enhanced CRTC2 expression drives malignant phenotypes and primary resistance to immunotherapy in hepatocellular carcinoma. iScience (2024) 27(6):109821. 10.1016/j.isci.2024.109821 38770131 PMC11103543

[76] Han SH Ko JY Jung S Oh S Kim DY Kang E VIM-AS1, which is regulated by CpG methylation, cooperates with IGF2BP1 to inhibit tumor aggressiveness *via* EPHA3 degradation in hepatocellular carcinoma. Exp Mol Med (2024) 56(12):2617–30. 10.1038/s12276-024-01352-6 39617786 PMC11671536

[77] Han S Fan H Zhong G Ni L Shi W Fang Y Nuclear KRT19 is a transcriptional corepressor promoting histone deacetylation and liver tumorigenesis. Hepatology (2025) 81(3):808–22. 10.1097/HEP.0000000000000875 38557414

[78] Rhee H Kim HY Choi JH Woo HG Yoo JE Nahm JH Keratin 19 expression in hepatocellular carcinoma is regulated by fibroblast-derived HGF *via* a MET-ERK1/2-AP1 and SP1 axis. Cancer Res (2018) 78(7):1619–31. 10.1158/0008-5472.CAN-17-0988 29363547

[79] Comet I Helin K . Revolution in the Polycomb hierarchy. Nat Struct Mol Biol (2014) 21(7):573–5. 10.1038/nsmb.2848 24992222

[80] Liu L Xu Z Zhong L Wang H Jiang S Long Q Enhancer of zeste homolog 2 (EZH2) promotes tumour cell migration and invasion *via* epigenetic repression of E-cadherin in renal cell carcinoma. BJU Int (2016) 117(2):351–62. 10.1111/bju.12702 24612432

[81] Sudo T Utsunomiya T Mimori K Nagahara H Ogawa K Inoue H Clinicopathological significance of EZH2 mRNA expression in patients with hepatocellular carcinoma. Br J Cancer (2005) 92(9):1754–8. 10.1038/sj.bjc.6602531 15856046 PMC2362028

[82] Dawson MA Kouzarides T . Cancer epigenetics: from mechanism to therapy. Cell (2012) 150(1):12–27. 10.1016/j.cell.2012.06.013 22770212

[83] Su C Zhang H Mo J Liao Z Zhang B Zhu P . SP1-activated USP27X-AS1 promotes hepatocellular carcinoma progression *via* USP7-mediated AKT stabilisation. Clin Transl Med (2024) 14(1):e1563. 10.1002/ctm2.1563 38279869 PMC10819096

[84] Tang L Ji Y Ni C Xu Z Shen Y Lu H EIF4A3-Mediated biogenesis of CircFADS1 promotes the progression of hepatocellular carcinoma *via* Wnt/β-Catenin pathway. Adv Sci (Weinh) (2025) 12(14):e2411869. 10.1002/advs.202411869 39965082 PMC11984884

[85] Koustas E Trifylli EM Sarantis P Papadopoulos N Papanikolopoulos K Aloizos G An Insight into the arising role of MicroRNAs in hepatocellular carcinoma: future diagnostic and therapeutic approaches. Int J Mol Sci (2023) 24(8):7168. 10.3390/ijms24087168 37108330 PMC10138911

[86] Shi T Morishita A Kobara H Masaki T . The role of long non-coding RNA and microRNA networks in hepatocellular carcinoma and its tumor microenvironment. Int J Mol Sci (2021) 22(19):10630. 10.3390/ijms221910630 34638971 PMC8508708

[87] Wang HC Yin WX Jiang M Han JY Kuai XW Sun R Function and biomedical implications of exosomal microRNAs delivered by parenchymal and nonparenchymal cells in hepatocellular carcinoma. World J Gastroenterol (2023) 29(39):5435–51. 10.3748/wjg.v29.i39.5435 37900996 PMC10600808

[88] Akkız H . Emerging role of cancer-associated fibroblasts in progression and treatment of hepatocellular carcinoma. Int J Mol Sci (2023) 24(4):3941. 10.3390/ijms24043941 36835352 PMC9964606

[89] Song M He J Pan QZ Yang J Zhao J Zhang YJ Cancer-Associated fibroblast-mediated cellular crosstalk supports hepatocellular carcinoma progression. Hepatology (2021) 73(5):1717–35. 10.1002/hep.31792 33682185

[90] Peng H Yang M Feng K Lv Q Zhang Y . Semaphorin 3C (Sema3C) reshapes stromal microenvironment to promote hepatocellular carcinoma progression. Signal Transduct Target Ther (2024) 9(1):169. 10.1038/s41392-024-01887-0 38956074 PMC11220018

[91] Mantovani A Marchesi F Malesci A Laghi L Allavena P . Tumour-associated macrophages as treatment targets in oncology. Nat Rev Clin Oncol (2017) 14(7):399–416. 10.1038/nrclinonc.2016.217 28117416 PMC5480600

[92] Murray PJ Allen JE Biswas SK Fisher EA Gilroy DW Goerdt S Macrophage activation and polarization: nomenclature and experimental guidelines. Immunity (2014) 41(1):14–20. 10.1016/j.immuni.2014.06.008 25035950 PMC4123412

[93] Sica A Mantovani A . Macrophage plasticity and polarization: *in vivo* veritas. J Clin Invest (2012) 122(3):787–95. 10.1172/JCI59643 22378047 PMC3287223

[94] Tang BF Xu WT Fang SJ Zhu JY Qiu RF Shen L MELK prevents radiofrequency ablation-induced immunogenic cell death and antitumor immune response by stabilizing FABP5 in hepatocellular malignancies. Mil Med Res (2025) 12(1):5. 10.1186/s40779-024-00588-7 39871325 PMC11773770

[95] Li X Yao W Yuan Y Chen P Li B Li J Targeting of tumour-infiltrating macrophages *via* CCL2/CCR2 signalling as a therapeutic strategy against hepatocellular carcinoma. Gut (2017) 66(1):157–67. 10.1136/gutjnl-2015-310514 26452628

[96] Tang B Zhu J Shi Y Wang Y Zhang X Chen B Tumor cell-intrinsic MELK enhanced CCL2-dependent immunosuppression to exacerbate hepatocarcinogenesis and confer resistance of HCC to radiotherapy. Mol Cancer (2024) 23(1):137. 10.1186/s12943-024-02049-0 38970074 PMC11225310

[97] Zhang Z Huang W Hu D Jiang J Zhang J Wu Z E-twenty-six-specific sequence variant 5 (ETV5) facilitates hepatocellular carcinoma progression and metastasis through enhancing polymorphonuclear myeloid-derived suppressor cell (PMN-MDSC)-mediated immunosuppression. Gut (2025). 10.1136/gutjnl-2024-333944 40015948

[98] Tu X Chen L Zheng Y Mu C Zhang Z Wang F S100A9(+)CD14(+) monocytes contribute to anti-PD-1 immunotherapy resistance in advanced hepatocellular carcinoma by attenuating T cell-mediated antitumor function. J Exp Clin Cancer Res (2024) 43(1):72. 10.1186/s13046-024-02985-1 38454445 PMC10921725

[99] Wong CC Wong CM . ETV5-S100A9 feed-forward loop connecting HCC and MDSCs to shape the immunosuppressive tumour microenvironment. Gut (2025). 10.1136/gutjnl-2025-335078 40147932

[100] Donne R Lujambio A . The liver cancer immune microenvironment: therapeutic implications for hepatocellular carcinoma. Hepatology (2023) 77(5):1773–96. 10.1002/hep.32740 35989535 PMC9941399

[101] Childs A Aidoo-Micah G Maini MK Meyer T . Immunotherapy for hepatocellular carcinoma. JHEP Rep (2024) 6(10):101130. 10.1016/j.jhepr.2024.101130 39308986 PMC11414669

[102] Chen J Feng W Sun M Huang W Wang G Chen X TGF-β1-Induced SOX18 elevation promotes hepatocellular carcinoma progression and metastasis through transcriptionally upregulating PD-L1 and CXCL12. Gastroenterology (2024) 167(2):264–80. 38417530 10.1053/j.gastro.2024.02.025

[103] Sun DY Wu WB Wu JJ Shi Y Xu JJ Ouyang SX Pro-ferroptotic signaling promotes arterial aging *via* vascular smooth muscle cell senescence. Nat Commun (2024) 15(1):1429. 10.1038/s41467-024-45823-w 38365899 PMC10873425

[104] Ma Y Wang J Liu L Zhu H Chen X Pan S Genistein potentiates the effect of arsenic trioxide against human hepatocellular carcinoma: role of Akt and nuclear factor-kappaB. Cancer Lett (2011) 301(1):75–84. 10.1016/j.canlet.2010.10.022 21078540

[105] Okano H Shiraki K Inoue H Kawakita T Saitou Y Enokimura N Fas stimulation activates NF-kappaB in SK-Hep1 hepatocellular carcinoma cells. Oncol Rep (2003) 10(5):1145–8. 12883671

[106] Song FN Duan M Liu LZ Wang ZC Shi JY Yang LX RANKL promotes migration and invasion of hepatocellular carcinoma cells *via* NF-kappaB-mediated epithelial-mesenchymal transition. PLoS One (2014) 9(9):e108507. 10.1371/journal.pone.0108507 25268581 PMC4182493

[107] Zhou Y Hu L Tang W Li D Ma L Liu H Hepatic NOD2 promotes hepatocarcinogenesis *via* a RIP2-mediated proinflammatory response and a novel nuclear autophagy-mediated DNA damage mechanism. J Hematol Oncol (2021) 14(1):9. 10.1186/s13045-020-01028-4 33413510 PMC7791875

[108] Xu Z Pei L Wang L Zhang F Hu X Gui Y . Snail1-dependent transcriptional repression of Cezanne2 in hepatocellular carcinoma. Oncogene (2014) 33(22):2836–45. 10.1038/onc.2013.243 23792447

[109] Du X Liu H Tian Z Zhang S Shi L Wang Y PI3K/AKT/mTOR pathway mediated-cell cycle dysregulation contribute to malignant proliferation of mouse spermatogonia induced by microcystin-leucine arginine. Environ Toxicol (2023) 38(2):343–58. 10.1002/tox.23691 36288207

[110] Yu JS Cui W . Proliferation, survival and metabolism: the role of PI3K/AKT/mTOR signalling in pluripotency and cell fate determination. Development (2016) 143(17):3050–60. 10.1242/dev.137075 27578176

[111] Yang J Pi C Wang G . Inhibition of PI3K/Akt/mTOR pathway by apigenin induces apoptosis and autophagy in hepatocellular carcinoma cells. Biomed Pharmacother (2018) 103:699–707. 10.1016/j.biopha.2018.04.072 29680738

[112] Gu D Ye M Zhu G Bai J Chen J Yan L Hypoxia upregulating ACSS2 enhances lipid metabolism reprogramming through HMGCS1 mediated PI3K/AKT/mTOR pathway to promote the progression of pancreatic neuroendocrine neoplasms. J Transl Med (2024) 22(1):93. 10.1186/s12967-024-04870-z 38263056 PMC10804556

[113] Karar J Maity A . PI3K/AKT/mTOR pathway in angiogenesis. Front Mol Neurosci (2011) 4:51. 10.3389/fnmol.2011.00051 22144946 PMC3228996

[114] Luo X Cao M Gao F He X . YTHDF1 promotes hepatocellular carcinoma progression *via* activating PI3K/AKT/mTOR signaling pathway and inducing epithelial-mesenchymal transition. Exp Hematol Oncol (2021) 10(1):35. 10.1186/s40164-021-00227-0 34088349 PMC8176587

[115] Sun EJ Wankell M Palamuthusingam P McFarlane C Hebbard L . Targeting the PI3K/Akt/mTOR pathway in hepatocellular carcinoma. Biomedicines (2021) 9(11):1639. 10.3390/biomedicines9111639 34829868 PMC8615614

[116] Su F Koeberle A . Regulation and targeting of SREBP-1 in hepatocellular carcinoma. Cancer Metastasis Rev (2024) 43(2):673–708. 10.1007/s10555-023-10156-5 38036934 PMC11156753

[117] Quan Z Peng B Hu K Liang L Liu M Liao L AP5Z1 affects hepatocellular carcinoma growth and autophagy by regulating PTEN ubiquitination and modulating the PI3K/Akt/mTOR pathway. J Transl Med (2025) 23(1):564. 10.1186/s12967-025-06537-9 40394639 PMC12090622

[118] Lei RE Shi C Zhang PL Hu BL Jiang HX Qin SY . IL-9 promotes proliferation and metastasis of hepatocellular cancer cells by activating JAK2/STAT3 pathway. Int J Clin Exp Pathol (2017) 10(7):7940–6. 31966644 PMC6965289

[119] Jiang H Wang Y Wen D Yu R Esa SS Lv K Targeting C21orf58 is a novel treatment strategy of hepatocellular carcinoma by disrupting the Formation of JAK2/C21orf58/STAT3 complex. Adv Sci (Weinh) (2024) 11(15):e2306623. 10.1002/advs.202306623 38342622 PMC11022693

[120] Li S Lin J Huang L Hu S Wang M Sun W STK31 drives tumor immune evasion through STAT3-IL-6 mediated CD8(+) T cell exhaustion. Oncogene (2025) 44(20):1452–62. 10.1038/s41388-024-03271-2 40025230

[121] Luo Y Lu J Lei Z Rao D Wang T Fu C GPR56 facilitates hepatocellular carcinoma metastasis by promoting the TGF-beta signaling pathway. Cell Death Dis (2024) 15(10):715. 10.1038/s41419-024-07095-6 39353900 PMC11445230

[122] Xu J Acharya S Sahin O Zhang Q Saito Y Yao J 14-3-3zeta turns TGF-beta's function from tumor suppressor to metastasis promoter in breast cancer by contextual changes of Smad partners from p53 to Gli2. Cancer Cell (2015) 27(2):177–92. 10.1016/j.ccell.2014.11.025 25670079 PMC4325275

[123] Liao Z Zhang H Liu F Wang W Liu Y Su C m(6)A-Dependent ITIH1 regulated by TGF-beta acts as a target for hepatocellular carcinoma progression. Adv Sci (Weinh) (2024) 11(42):e2401013. 10.1002/advs.202401013 39234824 PMC11558142

[124] Thangaraj JL Coffey M Lopez E Kaufman DS . Disruption of TGF-beta signaling pathway is required to mediate effective killing of hepatocellular carcinoma by human iPSC-derived NK cells. Cell Stem Cell (2024) 31(9):1327–43 e5. 10.1016/j.stem.2024.06.009 38986609 PMC11380586

[125] Qu P Wang Y Shao Z Lu D Mamiya T Wang W Primary cilium restricts TGF-beta/SMAD signaling induced RIBEs in the co-culture model. Cell Signal (2025) 134:111891. 10.1016/j.cellsig.2025.111891 40412783

[126] Mattu S Saliba C Sulas P Zavattari P Perra A Kowalik MA High frequency of beta-catenin mutations in Mouse hepatocellular Carcinomas induced by a nongenotoxic constitutive androstane receptor agonist. Am J Pathol (2018) 188(11):2497–507. 10.1016/j.ajpath.2018.07.022 30201494

[127] Adebayo Michael AO Ko S Tao J Moghe A Yang H Xu M Inhibiting glutamine-dependent mTORC1 activation ameliorates liver cancers driven by beta-catenin mutations. Cell Metab. (2019) 29(5):1135–50 e6. 30713111 10.1016/j.cmet.2019.01.002 PMC6506359

[128] Sun H Chen G Wen B Sun J An H Pang J Oligo-peptide I-C-F-6 inhibits hepatic stellate cell activation and ameliorates CCl(4)-induced liver fibrosis by suppressing NF-kappaB signaling and Wnt/beta-catenin signaling. J Pharmacol Sci (2018) 136(3):133–41. 10.1016/j.jphs.2018.01.003 29501581

[129] Berenguier C Chen X Allegrini B Guizouarn H Borgese F Etchebest C Cancer-associated loss-of-function mutations in KCNQ1 enhance Wnt/beta-catenin signalling disrupting epithelial homeostasis. Oncogene (2025). 10.1038/s41388-025-03447-4 40410368

[130] Moya IM Castaldo SA Van den Mooter L Soheily S Sansores-Garcia L Jacobs J Peritumoral activation of the Hippo pathway effectors YAP and TAZ suppresses liver cancer in mice. Science (2019) 366(6468):1029–34. 10.1126/science.aaw9886 31754005

[131] Zhang S Zhou D . Role of the transcriptional coactivators YAP/TAZ in liver cancer. Curr Opin Cell Biol (2019) 61:64–71. 10.1016/j.ceb.2019.07.006 31387016

[132] Li L Zhao GD Shi Z Qi LL Zhou LY Fu ZX . The Ras/Raf/MEK/ERK signaling pathway and its role in the occurrence and development of HCC. Oncol Lett (2016) 12(5):3045–50. 10.3892/ol.2016.5110 27899961 PMC5103898

[133] Samatar AA Poulikakos PI . Targeting RAS-ERK signalling in cancer: promises and challenges. Nat Rev Drug Discov (2014) 13(12):928–42. 10.1038/nrd4281 25435214

[134] Jin H Huang X Pan Q Ma N Xie X Wei Y The EIF3H-HAX1 axis increases RAF-MEK-ERK signaling activity to promote colorectal cancer progression. Nat Commun (2024) 15(1):2551. 10.1038/s41467-024-46521-3 38514606 PMC10957977

[135] Sanghvi VR Leibold J Mina M Mohan P Berishaj M Li Z The oncogenic action of NRF2 depends on De-glycation by Fructosamine-3-Kinase. Cell (2019) 178(4):807–19 e21. 10.1016/j.cell.2019.07.031 31398338 PMC6693658

[136] Tan CT Chang HC Zhou Q Yu C Fu NY Sabapathy K MOAP-1-mediated dissociation of p62/SQSTM1 bodies releases Keap1 and suppresses Nrf2 signaling. EMBO Rep (2021) 22(1):e50854. 10.15252/embr.202050854 33393215 PMC7788458

[137] Lu YY Zhu CY Ding YX Wang B Zhao SF Lv J Cepharanthine, a regulator of keap1-Nrf2, inhibits gastric cancer growth through oxidative stress and energy metabolism pathway. Cell Death Discov (2023) 9(1):450. 10.1038/s41420-023-01752-z 38086844 PMC10716385

[138] Guo C Ma Z Tao X Gao K Zhang W Wen A Therapeutic time window of Sodium of Danshensu on cerebral ischemia and its mechanism of inhibiting oxidative stress and ferroptosis through Nrf2 pathway. Brain Res Bull (2025) 227:111396. 10.1016/j.brainresbull.2025.111396 40403934

[139] Kusano H Ogasawara S Omuraya M Okudaira M Mizuochi S Mihara Y Sonic hedgehog expression in steatohepatitic hepatocellular carcinoma and its clinicopathological significance. Oncol Lett. (2024) 28(3):442. 10.3892/ol.2024.14575 39091582 PMC11292461

[140] Zheng X Zeng W Gai X Xu Q Li C Liang Z Role of the Hedgehog pathway in hepatocellular carcinoma (review). Oncol Rep (2013) 30(5):2020–6. 10.3892/or.2013.2690 23970376

[141] Li J He Y Cao Y Yu Y Chen X Gao X Upregulation of Twist is involved in Gli1 induced migration and invasion of hepatocarcinoma cells. Biol Chem (2018) 399(8):911–9. 10.1515/hsz-2018-0131 29908118

[142] Sridhar S Sharma I Sankpal UT Ghabach B Narra K Neerukonda L Targeted molecular therapeutic options for hepatocellular carcinoma. Crit Rev Oncog (2020) 25(1):47–55. 10.1615/CritRevOncog.2020034985 32865910 PMC11079775

[143] Cao LQ Xie Y Fleishman JS Liu X Chen ZS . Hepatocellular carcinoma and lipid metabolism: novel targets and therapeutic strategies. Cancer Lett. (2024) 597:217061. 10.1016/j.canlet.2024.217061 38876384

[144] Yu Y Shen X Xiao X Li L Huang Y . Butyrate modification promotes intestinal absorption and hepatic cancer cells targeting of ferroptosis inducer loaded nanoparticle for enhanced hepatocellular carcinoma therapy. Small (2023) 19(36):e2301149. 10.1002/smll.202301149 37165608

[145] Yao J Ning B Ding J . The gut microbiota: an emerging modulator of drug resistance in hepatocellular carcinoma. Gut Microbes (2025) 17(1):2473504. 10.1080/19490976.2025.2473504 40042184 PMC11901387

[146] Kudo M Finn RS Qin S Han KH Ikeda K Piscaglia F Lenvatinib *versus* sorafenib in first-line treatment of patients with unresectable hepatocellular carcinoma: a randomised phase 3 non-inferiority trial. Lancet (2018) 391(10126):1163–73. 10.1016/S0140-6736(18)30207-1 29433850

[147] Ye X Fang X Li F Jin D . Targeting TIME in advanced hepatocellular carcinoma: mechanisms of drug resistance and treatment strategies. Crit Rev Oncol Hematol. (2025) 211:104735. 10.1016/j.critrevonc.2025.104735 40250780

[148] Yang G Ren Y Li Y Tang Y Yuan F Cao M Post-treatment adverse events ranking in targeted immunotherapy for hepatocellular carcinoma: a network meta-analysis based on risk probability assessment. Crit Rev Oncol Hematol. (2025) 211:104737. 10.1016/j.critrevonc.2025.104737 40252815

[149] Hu B Zou T Qin W Shen X Su Y Li J Inhibition of EGFR overcomes acquired Lenvatinib resistance driven by STAT3-ABCB1 signaling in hepatocellular carcinoma. Cancer Res (2022) 82(20):3845–57. 10.1158/0008-5472.CAN-21-4140 36066408 PMC9574378

[150] Al-Salama ZT Syed YY Scott LJ . Lenvatinib: a review in hepatocellular carcinoma. Drugs (2019) 79(6):665–74. 10.1007/s40265-019-01116-x 30993651

[151] Sangro B Sarobe P Hervás-Stubbs S Melero I . Advances in immunotherapy for hepatocellular carcinoma. Nat Rev Gastroenterol Hepatol (2021) 18(8):525–43. 10.1038/s41575-021-00438-0 33850328 PMC8042636

[152] El-Khoueiry AB Sangro B Yau T Crocenzi TS Kudo M Hsu C Nivolumab in patients with advanced hepatocellular carcinoma (CheckMate 040): an open-label, non-comparative, phase 1/2 dose escalation and expansion trial. Lancet (2017) 389(10088):2492–502. 10.1016/S0140-6736(17)31046-2 28434648 PMC7539326

[153] Zhu AX Finn RS Edeline J Cattan S Ogasawara S Palmer D Pembrolizumab in patients with advanced hepatocellular carcinoma previously treated with sorafenib (KEYNOTE-224): a non-randomised, open-label phase 2 trial. Lancet Oncol (2018) 19(7):940–52. 10.1016/S1470-2045(18)30351-6 29875066

[154] Cheng AL Qin S Ikeda M Galle PR Ducreux M Kim TY Updated efficacy and safety data from IMbrave150: atezolizumab plus bevacizumab vs. sorafenib for unresectable hepatocellular carcinoma. J Hepatol (2022) 76(4):862–73. 10.1016/j.jhep.2021.11.030 34902530

[155] Hegde PS Wallin JJ Mancao C . Predictive markers of anti-VEGF and emerging role of angiogenesis inhibitors as immunotherapeutics. Semin Cancer Biol (2018) 52(Pt 2):117–24. 10.1016/j.semcancer.2017.12.002 29229461

[156] Zhou Y Wei S Xu M Wu X Dou W Li H CAR-T cell therapy for hepatocellular carcinoma: current trends and challenges. Front Immunol (2024) 15:1489649. 10.3389/fimmu.2024.1489649 39569202 PMC11576447

[157] Zhou F Shang W Yu X Tian J . Glypican-3: a promising biomarker for hepatocellular carcinoma diagnosis and treatment. Med Res Rev (2018) 38(2):741–67. 10.1002/med.21455 28621802

[158] Yu B Ma W . Biomarker discovery in hepatocellular carcinoma (HCC) for personalized treatment and enhanced prognosis. Cytokine Growth Factor Rev (2024) 79:29–38. 10.1016/j.cytogfr.2024.08.006 39191624

[159] Perica K Varela JC Oelke M Schneck J . Adoptive T cell immunotherapy for cancer. Rambam Maimonides Med J. (2015) 6(1):e0004. 10.5041/RMMJ.10179 25717386 PMC4327320

[160] Schiller JT Lowy DR . Understanding and learning from the success of prophylactic human papillomavirus vaccines. Nat Rev Microbiol (2012) 10(10):681–92. 10.1038/nrmicro2872 22961341 PMC6309166

[161] Sahin U Türeci Ö . Personalized vaccines for cancer immunotherapy. Science (2018) 359(6382):1355–60. 10.1126/science.aar7112 29567706

[162] Zuo B Zhang Y Zhao K Wu L Qi H Yang R Universal immunotherapeutic strategy for hepatocellular carcinoma with exosome vaccines that engage adaptive and innate immune responses. J Hematol Oncol (2022) 15(1):46. 10.1186/s13045-022-01266-8 35488312 PMC9052531

[163] Llovet JM Pinyol R Yarchoan M Singal AG Marron TU Schwartz M Adjuvant and neoadjuvant immunotherapies in hepatocellular carcinoma. Nat Rev Clin Oncol (2024) 21(4):294–311. 10.1038/s41571-024-00868-0 38424197 PMC11984461

[164] Feng T Xie F Lyu Y Yu P Chen B Yu J The arginine metabolism and its deprivation in cancer therapy. Cancer Lett. (2025) 620:217680. 10.1016/j.canlet.2025.217680 40157492

[165] Cheng PN Leung YC Lo WH Tsui SM Lam KC . Remission of hepatocellular carcinoma with arginine depletion induced by systemic release of endogenous hepatic arginase due to transhepatic arterial embolisation, augmented by high-dose insulin: arginase as a potential drug candidate for hepatocellular carcinoma. Cancer Lett (2005) 224(1):67–80. 10.1016/j.canlet.2004.10.050 15911102

[166] Guerrini G Magrì D Gioria S Medaglini D Calzolai L . Characterization of nanoparticles-based vaccines for COVID-19. Nat Nanotechnol (2022) 17(6):570–6. 10.1038/s41565-022-01129-w 35710950

[167] Wang B Hu S Teng Y Chen J Wang H Xu Y Current advance of nanotechnology in diagnosis and treatment for malignant tumors. Signal Transduct Target Ther (2024) 9(1):200. 39128942 10.1038/s41392-024-01889-y PMC11323968

[168] Yan Y Liu XY Lu A Wang XY Jiang LX Wang JC . Non-viral vectors for RNA delivery. J Control Release (2022) 342:241–79. 10.1016/j.jconrel.2022.01.008 35016918 PMC8743282

[169] Zhang Y Luo J Gui X Zheng Y Schaar E Liu G Bioengineered nanotechnology for nucleic acid delivery. J Control Release (2023) 364:124–41. 10.1016/j.jconrel.2023.10.034 37879440 PMC10838211

[170] Zhu X Tao W Liu D Wu J Guo Z Ji X Surface De-PEGylation controls nanoparticle-mediated siRNA delivery *in vitro* and *in vivo* . Theranostics (2017) 7(7):1990–2002. 10.7150/thno.18136 28638484 PMC5479285

[171] Zhou S Ma Y Liu X Yu P Huang N Song L Targeted delivery of Glypican 3 (GPC3) antibody-modified MicroRNA (miR let-7b-5p) polymer nanoparticles to Sorafenib-Resistant hepatsocellular carcinoma cells. J Biomed Nanotechnol (2021) 17(4):677–90. 10.1166/jbn.2021.3033 35057893

[172] Yuan Y Sun W Xie J Zhang Z Luo J Han X RNA nanotherapeutics for hepatocellular carcinoma treatment. Theranostics (2025) 15(3):965–92. 10.7150/thno.102964 39776807 PMC11700867

[173] Liu B Cao W Qiao G Yao S Pan S Wang L Effects of gold nanoprism-assisted human PD-L1 siRNA on both gene down-regulation and photothermal therapy on lung cancer. Acta Biomater (2019) 99:307–19. 10.1016/j.actbio.2019.08.046 31513911

[174] Li S Saw PE Lin C Nie Y Tao W Farokhzad OC Redox-responsive polyprodrug nanoparticles for targeted siRNA delivery and synergistic liver cancer therapy. Biomaterials (2020) 234:119760. 10.1016/j.biomaterials.2020.119760 31945619

[175] Sarker D Plummer R Meyer T Sodergren MH Basu B Chee CE MTL-CEBPA, a small activating RNA therapeutic upregulating C/EBP-α, in patients with advanced liver cancer: a First-in-Human, multicenter, Open-Label, phase I trial. Clin Cancer Res. (2020) 26(15):3936–46. 10.1158/1078-0432.CCR-20-0414 32357963

[176] Jin T Luo M Chen F Bai J Ding J . Harnessing the power of AI for enhanced diagnosis and treatment of hepatocellular carcinoma. Turk J Gastroenterol (2024) 36(4):200–8. 10.5152/tjg.2024.24325 39714118 PMC12001482

[177] Calderaro J Seraphin TP Luedde T Simon TG . Artificial intelligence for the prevention and clinical management of hepatocellular carcinoma. J Hepatol (2022) 76(6):1348–61. 10.1016/j.jhep.2022.01.014 35589255 PMC9126418

[178] Santarpia M Liguori A D'Aveni A Karachaliou N Gonzalez-Cao M Daffinà MG Liquid biopsy for lung cancer early detection. J Thorac Dis (2018) 10(Suppl. 7):S882–s97. 10.21037/jtd.2018.03.81 29780635 PMC5945693

[179] Luk JM Lam BY Lee NP Ho DW Sham PC Chen L Artificial neural networks and decision tree model analysis of liver cancer proteomes. Biochem Biophys Res Commun (2007) 361(1):68–73. 10.1016/j.bbrc.2007.06.172 17644064

[180] Larson JA Johnson MH Bhayani SB . Application of surgical safety standards to robotic surgery: five principles of ethics for nonmaleficence. J Am Coll Surg (2014) 218(2):290–3. 10.1016/j.jamcollsurg.2013.11.006 24315652

[181] Leonard S Wu KL Kim Y Krieger A Kim PC . Smart tissue anastomosis robot (STAR): a vision-guided robotics system for laparoscopic suturing. IEEE Trans Biomed Eng (2014) 61(4):1305–17. 10.1109/TBME.2014.2302385 24658254

[182] Di Benedetto F Petrowsky H Magistri P Halazun KJ . Robotic liver resection: hurdles and beyond. Int J Surg (2020) 82s:155–62. 10.1016/j.ijsu.2020.05.070 32504813

[183] Levi Sandri GB de Werra E Mascianà G Colasanti M Santoro R D'Andrea V Laparoscopic and robotic approach for hepatocellular carcinoma-state of the art. Hepatobiliary Surg Nutr (2016) 5(6):478–84. 10.21037/hbsn.2016.05.05 28124002 PMC5218911

